# Transcription-dependent and -independent functions of *Drosophila* p53 isoforms in the induction of apoptosis and senescence-associated tumorigenesis

**DOI:** 10.1038/s41419-026-08571-x

**Published:** 2026-03-25

**Authors:** Marina Pérez-Aguilera, Mireya Ruiz-Losada, Paula Gil Cortes, Marian Benchaib, Carlos Rubio, Luis Alberto Baena-López, Antonio Baonza, Carlos Estella

**Affiliations:** 1 Centro de Biología Molecular Severo Ochoa (CBM), CSIC-UAM, Nicolás Cabrera 1, Universidad Autónoma de Madrid, Cantoblanco, Madrid, Spain; 2 Department of Biology, University of Konstanz, Konstanz, Germany; 3 Present Address: Department of Medical Oncology, Lymphoma Research Group, Hospital Universitario Puerta de Hierro-Majadahonda, IDIPHISA, Madrid, Spain

**Keywords:** Developmental biology, Senescence, Apoptosis

## Abstract

The tumor suppressor p53 orchestrates critical cellular responses to stress, including cell cycle arrest, DNA repair, senescence, and apoptosis. While extensive research has elucidated many aspects of p53 function, the isoform-specific mechanisms governing cell fate decisions remain incompletely understood. Here, we leverage the simplified *p53* gene architecture in *Drosophila* to systematically dissect the apoptotic and tumorigenic potential of individual p53 isoforms, uncovering fundamental differences in their function. Our findings indicate that whereas p53-A and p53-E pro-apoptotic activity strictly depends on the proliferative state of the cell, the full-length p53-B isoform -structurally analogous to vertebrate p53- induces apoptosis independently of cell cycle status. Furthermore, p53-B triggers apoptosis via transcription-independent mechanisms involving direct activation of the initiator caspase Dronc. We also show that all isoforms promote tumorigenesis by inducing the JNK pathway and the formation of senescent cells through distinct mechanisms in cells that are unable to complete the apoptosis program. Importantly, some of these findings are largely recapitulated by human versions of p53 when ectopically expressed in *Drosophila* cells. Together our data, reveal that p53 isoforms govern apoptosis and senescence-associated tumorigenesis through distinct molecular mechanisms, providing new insights into the complexity of p53-mediated cell fate determination.

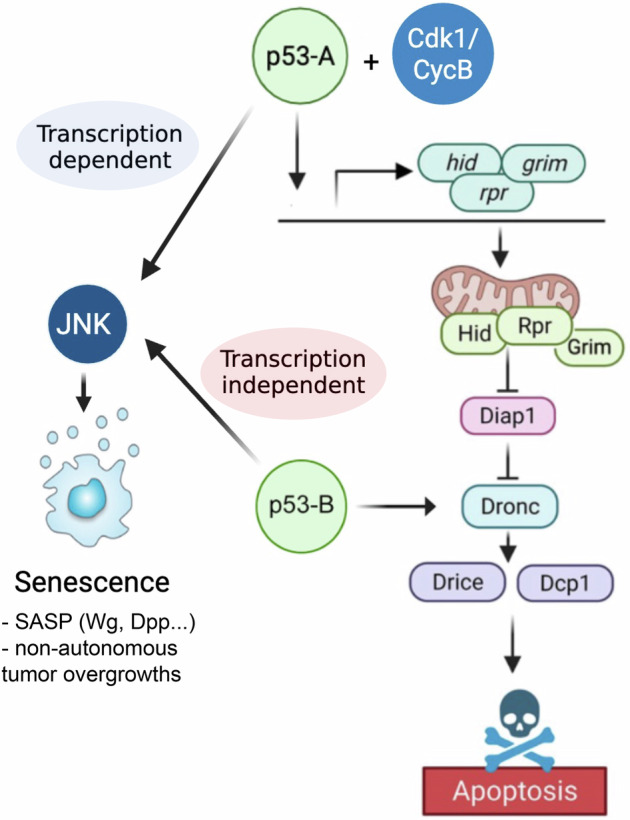

## Introduction

In response to cellular stress, such as DNA damage, the transcription factor p53 is activated promoting many different responses, such as cell cycle arrest, DNA repair, senescence or apoptosis that ultimately determine the survival or the death of the cell [1, 2]. Accordingly, defects in the coordination of these responses are a major cause of tumorigenesis and *p53* mutations are common in many cancers, underscoring its importance as a tumor suppressor [1]. Many of these mutations produce p53 variants that not only lose wild-type function but also exert dominant-negative effects on the remaining wild-type protein [3]. Notably, mutant p53 can acquire oncogenic properties that extend beyond simple p53 inactivation [4, 5]. Many mechanisms have been proposed that partially explain how p53 regulates different transcriptional programs [6]. These include p53 post-translational modifications, protein-protein interactions, affinity to p53 responsive elements (REs) and p53 levels among others [2, 7, 8]. In addition, the developmental and cellular context, such as the proliferative and differentiation state of the cell can influence p53 cell fate decisions [9–13]. To add further complexity, p53 in humans encodes 12 isoforms due to the use of alternative promoters, splicing sites and initiation codons whose functions in development and tumor progression remain poorly understood [14, 15]. Aberrant expression of these isoforms and their interactions with each other and with other cellular proteins have been linked not only to p53’s tumor suppressor role, but also to its oncogenic potential [16, 17]. Different protein isoforms are generated through various combinations of an N-terminal transactivation domain (TAD), a central DNA-binding domain (DBD), and a C-terminal oligomerization domain (OD), resulting in distinct N- and C-terminal regions that influence their regulatory functions and interactions with other proteins [14].

p53 exhibits remarkable structural and functional conservation between vertebrates and *Drosophila melanogaster*, particularly in its role as a transcription factor mediating the apoptotic response to DNA damage [18–22]. This makes *Drosophila* a powerful and genetically tractable model for dissecting p53-regulated pathways and understanding tumor suppression mechanisms [23–26]. However, in contrast to its vertebrate orthologue, *Drosophila* p53 is not required for the cell cycle arrest after irradiation [18, 19]. *Drosophila* has a single p53 orthologue that encodes only three distinct protein isoforms (A, B, and E), providing a simplified system to study conserved roles of p53 in stress responses and tissue homeostasis [24, 26]. *Drosophila* p53 isoforms may have specific functions during development and in response to DNA damage [27–34]. In this regard, our recent studies have shown that the pro-apoptotic activity of p53-A in response to DNA damage is modulated by the proliferative state of the cell [13]. Specifically, we identified a direct interaction between the main *Drosophila* p53 isoform (p53-A) and cyclin-dependent kinase 1 (Cdk1), the key regulator of the G2/M phase transition [13, 23]. We have also reported that p53-A can induce a senescence-like state that promotes tumorigenesis in cells unable to complete the apoptotic program [35].

Here, we systematically characterized the functional roles of *Drosophila* p53 isoforms in apoptotic induction and the formation of senescence-associated tumors. Our results show that, unlike p53-A and p53-E, the p53-B isoform induces apoptosis independently of cell cycle status. Mechanistically, this apoptotic activity is explained by p53-B’s unique ability to physically interact with the initiator caspase Dronc, thereby promoting its activation in a transcription independent manner. In parallel, our new studies show that all p53 isoforms have tumorigenic potential in cells unable to implement the apoptosis program by inducing the activation of the JNK pathway and the formation of senescent cells. Importantly, as an indication of evolutionary conservation, we also found that apoptosis induction by the expression of human p53 in *Drosophila* cells displays partial cell cycle dependence and can trigger cell death through a transcription-independent mechanism.

## Results

### Cell cycle-dependent regulation of the pro-apoptotic activity of the different p53 isoforms

**Fig. 1:**
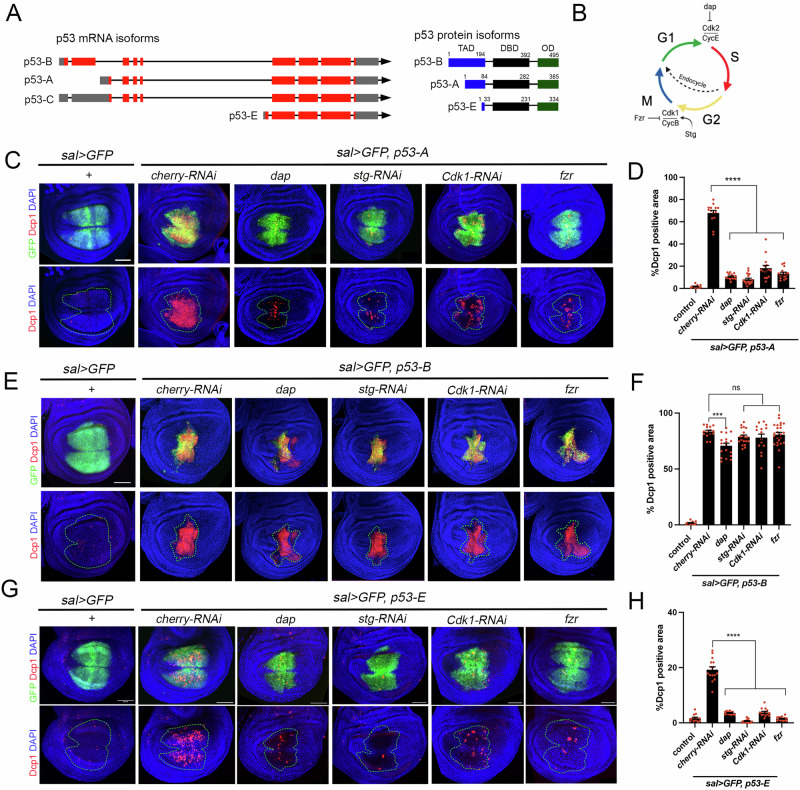
Apoptotic induction by p53 isoforms is differentially regulated by the cell cycle. **A** Diagram showing the different p53 isoforms and their respective proteins. Note that p53-C and p53-A encode the same protein. Red boxes represent exons, while blue, black and green boxes denote the different protein domains. TAD-transactivation domain, DBD-DNA binding domain, and OD-oligomerization domain. The amino acid positions that define the p53 domains are indicated. **B** Schematic representation of the cell cycle and the different genetic tools used to block it. **C**,**E**,**G** Wing imaginal discs expressing the corresponding transgenes under the *sal* > *GFP* driver. Imaginal discs were stained for Dcp1 (red), GFP (green) and DAPI (blue). A dotted green line marks the *sal* domain. The scale bar is 50 μm. **D**,**F**,**H** Quantification of Dcp1 staining in the *sal* domain of wing imaginal discs from the genotypes presented in (**C**), (**E**), and (**G**). The data are derived from three independent biological replicates, analyzing more than 13 discs per genotype. Error bars indicate SEM. ****P value < 0.0001 and ***P value < 0.005 and not significant (ns) *P* value > 0.05 by one-way ANOVA.

In *Drosophila* the p53 locus encodes for three different protein isoforms, A, B, and E, which share the DNA binding domain and the C-terminus but differ in the length of the TAD at the amino terminus [24, 27] (Fig. 1A). We have previously shown that the pro-apoptotic activity of *Drosophila* p53-A is regulated by the proliferative state of the cell [13]. To assess the apoptotic potential of each *Drosophila* p53 isoform during distinct phases of the cell cycle, we used transgenic flies expressing Myc-tagged versions of each isoform under UAS control. All constructs were inserted at the same chromosomal location to ensure consistent expression levels [27]. We then expressed each isoform individually in wing discs using the *spalt*^*EPV*^*-Gal4* driver (*sal* > *GFP*), while simultaneously manipulating the cell cycle. Specifically, we arrested cells in G1 by co-expressing *dacapo* (Dap/p21), or in G2 by using RNA interference against *string* (Stg/Cdc25) or Cdk1, within the same *sal* > *GFP* domain. In a similar set of experiments, we ectopically expressed *fizzy related* (Fzr/Cdh1) to switch the mitotic cycle to an endocycle (Fig. 1B). The endocycle is particularly relevant because it alternates between G and S phases without entering mitosis, which is achieved by downregulating Cdk1 activity [36]. Consistent with our previous results [13], the ability of p53-A to induce apoptosis is strongly suppressed in cell cycle-arrested cells and endocycle-induced cells, as visualized by staining for the active effector caspase Dcp1 (Fig. 1C, D). The longer isoform, p53-B, induces a more robust apoptotic response than the other isoforms, and importantly, this pro-apoptotic activity is independent of the proliferative status of the cell (Fig. 1E, F). The shorter isoform, p53-E also induces apoptosis, but to a lesser extent than p53-A or p53-B, and in a cell cycle dependent manner (Fig. 1G, H).

To further validate these results, we examined the apoptotic induction capabilities of p53-A and p53-B isoforms when ectopically expressed in the endocycling cells of the salivary glands (Fig. S1). Although p53-A is being highly expressed in salivary gland cells, it fails to induce apoptosis in these endocycling cells (Fig. S1B). In stark contrast, p53-B triggers robust Dcp-1 immunoreactivity, as an indication of apoptosis (Fig. S1C). These observations strongly suggest that the regulation of p53-induced apoptosis by the cell cycle is isoform-specific, with p53-B possibly triggering apoptosis through different molecular mechanisms to p53-A.

### p53-B can induce apoptosis independently of the pro-apoptotic genes

**Fig. 2:**
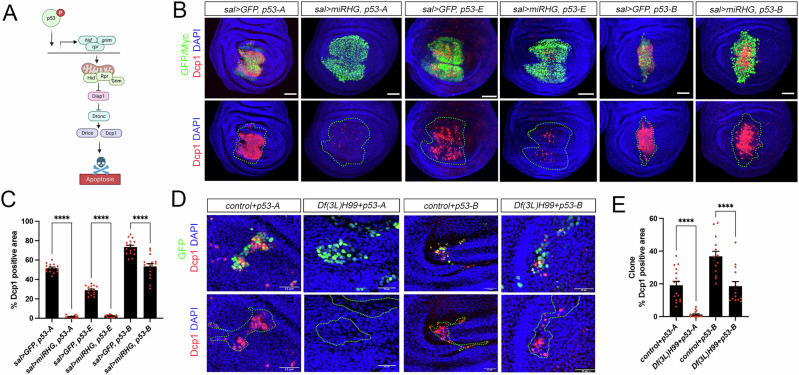
p53-B induces apoptosis independently of the RHG genes. **A** A simplified representation of the apoptotic pathway triggered by p53. Created with BioRender.com. **B** Wing imaginal discs expressing the corresponding transgenes under the *sal* > *GFP* driver. Imaginal discs were stained for Dcp1 (red), GFP or Myc as indicated (green) and DAPI (blue). A dotted green line marks the *sal* domain. Note that the expression of the UAS-*miRHG* strongly reduces the apoptotic induction by p53-A and p53-E, but not by p53-B. Scale bar is 50 μm. **C** Quantification of Dcp1 staining in the *sal* domain of wing imaginal discs from the genotypes presented in (**B**). The data are derived from three independent biological replicates, analyzing more than 14 discs per genotype. Error bars indicate SEM. ****P value < 0.0001 by one-way ANOVA. **D** Representative images of GFP-marked control clones and clones homozygous for *Df(3* *L)H99* that simultaneously expressed either *p53-A* or *p53-B* in wing imaginal discs stained for Dcp1 (red), GFP (green), and DAPI (blue). A dotted green line marks the clone boundary. The scale bar is 25 μm. **E** Quantification of Dcp1 staining within the GFP-marked clones from the genotypes presented in (**D**). Error bars indicate SEM. *n* > 14 disc per genotype. ****P value < 0.0001 by one-way ANOVA.

p53 encodes a transcription factor that binds directly to the regulatory regions of the pro-apoptotic genes *reaper* (*rpr*) and *head involution defective* (*hid*) [18, 23, 27] (Fig. 2A). Given that all isoforms retain the full DBD, we sought to determine whether each p53 isoform induces apoptosis through transcriptional activation of pro-apoptotic target genes. To this end, we simultaneously inhibited the function of Rpr, Hid, and Grim (RHG) by overexpressing a microRNA (UAS*-miRHG*) that targets their expression in cells expressing the different p53 isoforms. Strikingly, these experiments revealed that knockdown of pro-apoptotic genes significantly reduced apoptosis induced by p53-A and p53-E, but had much less effect on p53-B–mediated cell death (Fig. 2B, C).

To confirm that p53-B can induce apoptosis independently of RHG, we expressed this isoform in cells homozygous for a deficiency that completely deletes these genes (Df(3L)H99) [37]. As a control, we performed the same experiment overexpressing *p53-A*. Consistent with our previous findings, cells mutant for the RHG genes only prevented the cell death induced by p53-A (Fig. 2D, E), thus confirming that the p53-B isoform can trigger apoptosis independently of the pro-apoptotic genes.

Activation of the c-Jun N-terminal kinase (JNK) pathway induces apoptosis in response to cellular stress and DNA damage by promoting the expression of pro-apoptotic genes [23, 38–40] (Fig. S2A). Consistent with the ability of p53 to activate JNK signaling [38, 39], we observed that all p53 isoforms trigger the expression of the JNK transcriptional reporter *TRE-RFP* (Fig. S2B–E). However, whereas JNK activation mediated by p53-A depends on the proliferative status, p53-B is able to induce the *TRE-RFP* activation even in cell cycle arrested cells (Fig. S2B–F). We confirmed that JNK-induced cell death depends on pro-apoptotic genes, as expression of a constitutively active form of Hemipterous (Hep^CA^) induced strong apoptosis that was completely suppressed by knocking down RHG (Fig. S2G, H). We then tested whether the induction of apoptosis by p53-B requires JNK activity by co-expressing p53-B with a dominant-negative form of the Jun Kinase Basket (Bsk^DN^). Only a modest reduction in cell death was observed, suggesting that p53-B can induce apoptosis through molecular mechanisms largely independently of the JNK pathway and of the pro-apoptotic genes (Fig. S2I, J).

### p53-B promotes apoptosis by enhancing Dronc activation

**Fig. 3:**
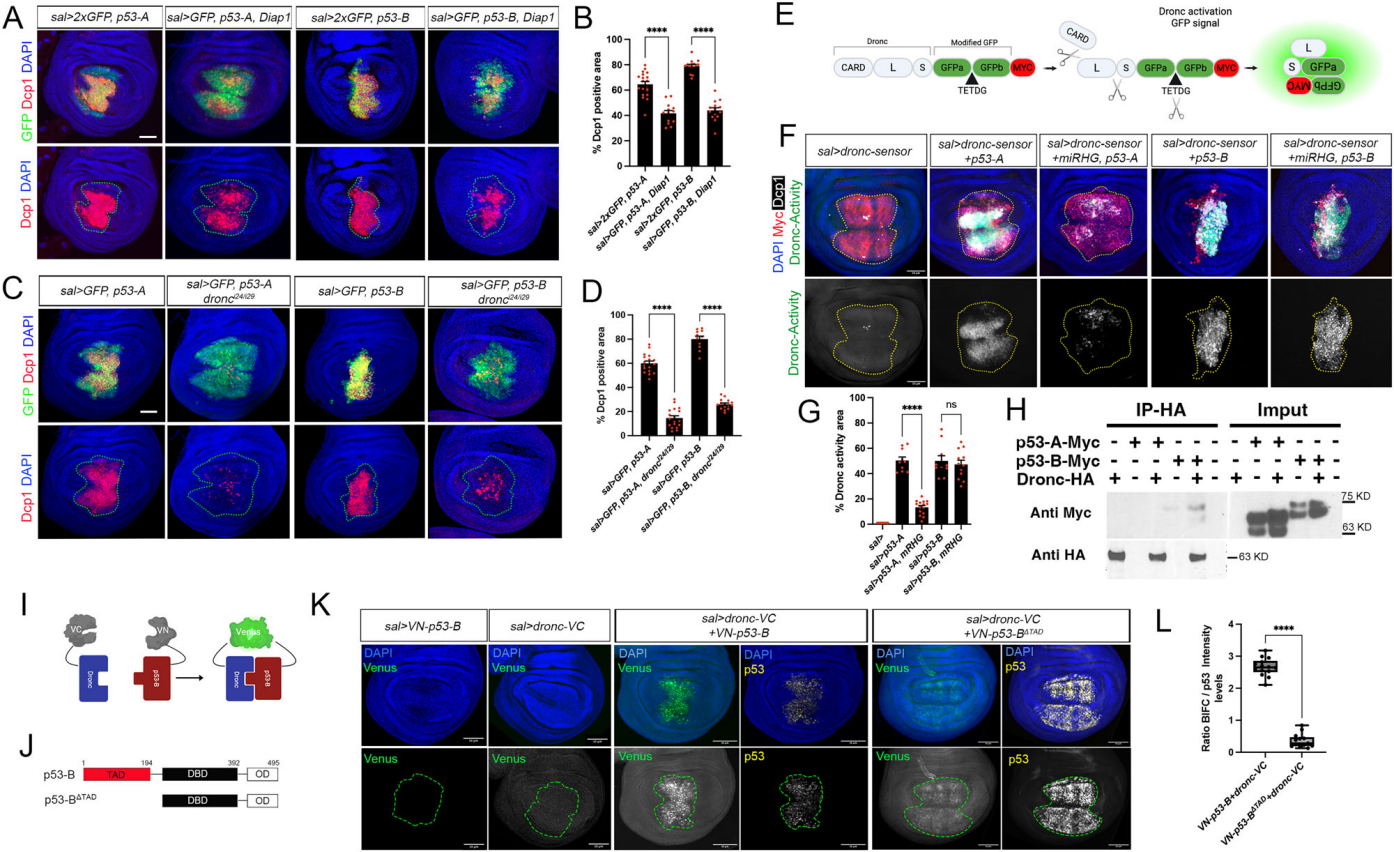
p53-B promotes apoptosis by enhancing Dronc activation. **A**,**C** Wing imaginal discs expressing the corresponding transgenes under the *sal* > *GFP* driver. Where indicated p53-A and p53-B expression was performed in a *dronc* mutant background. Imaginal discs were stained for Dcp1 (red), GFP (green) and DAPI (blue). A dotted green line marks the *sal* domain. The scale bar is 50 μm. **B**,**D** Quantification of Dcp1 staining in the *sal* domain of wing imaginal discs from the genotypes presented in (**A**) and (**C**). The data are derived from three independent biological replicates, analyzing more than 13 discs per genotype. Error bars indicate SEM. ****P value < 0.0001 by one-way ANOVA. **E** Cartoon of the Dronc activity sensor. The modified GFP includes a Dronc-specific cleavage site (TETDG) that when cleaved triggers a conformational change in the protein that leads to the emission of green fluorescence. The different Dronc domains are indicated as CARD, large (L) and small (S) subunits. **F** Wing imaginal discs expressing the Dronc activity sensor under the *sal>* driver and the corresponding transgenes. The imaginal discs were stained for GFP (green), Dcp1 (white), Myc (red) and DAPI (blue). A dotted yellow line marks the sal domain delimited by Myc staining. The scale bar is 50 μm. **G** Quantification of GFP (Dronc activity) in the *sal* domain of wing imaginal discs from the genotypes presented in (**F**). The data are derived from three independent biological replicates, analyzing more than ten discs per genotype Error bars indicate SEM. ****P value < 0.0001 and not significant (ns) *P* value > 0.05 by one-way ANOVA. **H** Wing discs lysates were immunoprecipitated (IP) using anti- HA conjugated to Protein A/G magnetic beads. Input (50% of lysate) and IP eluates were resolved by SDS-PAGE and probed with primary antibody against Myc and HA. **I** Cartoon illustrating the BiFC principle. No fluorescent N- and C-terminal fragments of the GFP variant Venus are fused to p53-B (VN-p53) and Dronc (Dronc-VC). When both proteins are co-expressed and interact, the Venus fragment is reconstructed and emits green fluorescence. This technique enables direct protein interactions to be visualized in living cells. **J** Scheme showing the different versions of the p53-B protein generated for the BiFC analysis. Amino acid positions defining p53 domains are indicated. **K** BiFC analysis by the co-expression of VN-p53-B, Dronc-VC, Dronc-VC and VN-p53-B and or Dronc-VC and VN-p53-B^ΔTAD^ with the *sal> driver*. A dotted green line marks the *sal* domain delimited by p53 staining. The scale bar is 50 μm. **L** Quantification of Venus intensity in the *sal* domain of wing imaginal discs from the genotypes presented in (**K**), expressed as a ratio between Venus signal and p53 staining intensity levels. The data are derived from three independent biological replicates, analyzing more than 13 discs per genotype. Error bars indicate standard deviation. ****P value < 0.0001 by one-way ANOVA.

Next, we investigated the potential molecular mechanisms that enable p53-B-dependent apoptosis. In response to an apoptotic stimulus, RHG proteins downregulate the inhibitor of apoptosis Diap1, facilitating the activation of the initiator caspase Dronc [41](Fig. 2A). To test whether p53-B requires Dronc activity to trigger cell death, we first co-expressed p53-A and p53-B isoforms with the Dronc inhibitor Diap1. We found that Diap1 can moderately suppress the apoptotic induction of both p53-A, and p53-B (Fig. 3A, B). To confirm this result, we expressed *p53-A* and *p53-B* in a *dronc*^*i24/i29*^ background, which has a complete lack of function for the apical caspase Dronc. The induction of apoptosis by both isoforms was strongly suppressed in the absence of Dronc (Fig. 3C, D). To assess the ability of p53 isoforms to activate Dronc and its dependency on the expression of pro-apoptotic genes, we manipulated their expression in RHG-deficient cells that also expressed a Dronc activation sensor. This sensor is a UAS construct that encodes a wild type form of Dronc fused to a modified Myc-tagged GFP (see Material and Methods). The modified GFP contains an intrinsic Dronc-specific cleavage site (TETDG), which, upon excision, induces a conformational rearrangement that results in green fluorescent emission (Fig. 3E). As predicted, the expression of *p53-A* resulted in a significant activation of the Dronc sensor. However, this effect was blocked when the apoptotic pathway was hindered at the level of the RHG genes (Fig. 3F, G). In stark contrast, activation of the Dronc sensor by p53-B remained unaffected by knocking down the pro-apoptotic genes (Fig. 3F, G). These results confirmed that p53-A and p53-B induce apoptosis via distinct molecular mechanisms. They also suggest that p53-B may directly enhance Dronc activation, raising the possibility of a direct molecular interaction between the two proteins.

To assess the potential interaction between p53-B and Dronc, we used two different approaches. First, we performed a co-immunoprecipitation assay using wing imaginal discs that expressed an endogenous HA-tagged version of Dronc, as well as UAS Myc-tagged versions of p53-A or p53-B, which were driven by *sal-Gal4*. Our results show that Dronc co-immunoprecipitated with p53-B but not with p53-A (Fig. 3H), suggesting a specific interaction between Dronc and the p53-B isoform. Secondly, we used a bimolecular fluorescence complementation (BiFC) assay, which relies on the reconstruction of the Venus fluorescent protein when two non-fluorescent Venus fragments, each fused to a protein of interest, interact (in this case UAS-*dronc-VC* and UAS-*VN-p53-B*) (Fig. 3I) [42]. While the expression of each construct alone did not result in a BiFC signal, the co-expression of *dronc-VC* and *VN-p53-B* did result in a detectable nuclear Venus signal, indicating a specific interaction in vivo (Fig. 3I–K). Importantly, the co-expression of Dronc-VC with a TAD-deleted variant of p53-B (VN-p53-B^ΔTAD^), did not result in Venus reconstitution and fluorescent signal (Fig. 3J–L), suggesting that the TAD is required for the p53-B-Dronc interaction.

Collectively, our results suggest that the p53-A isoform regulates apoptosis by activating the transcription of pro-apoptotic genes. In contrast, the p53-B isoform may directly activate the apoptotic pathway at the level of Dronc and independently of the RHG genes.

### p53-B can induce apoptosis in a transcriptional independent manner

**Fig. 4:**
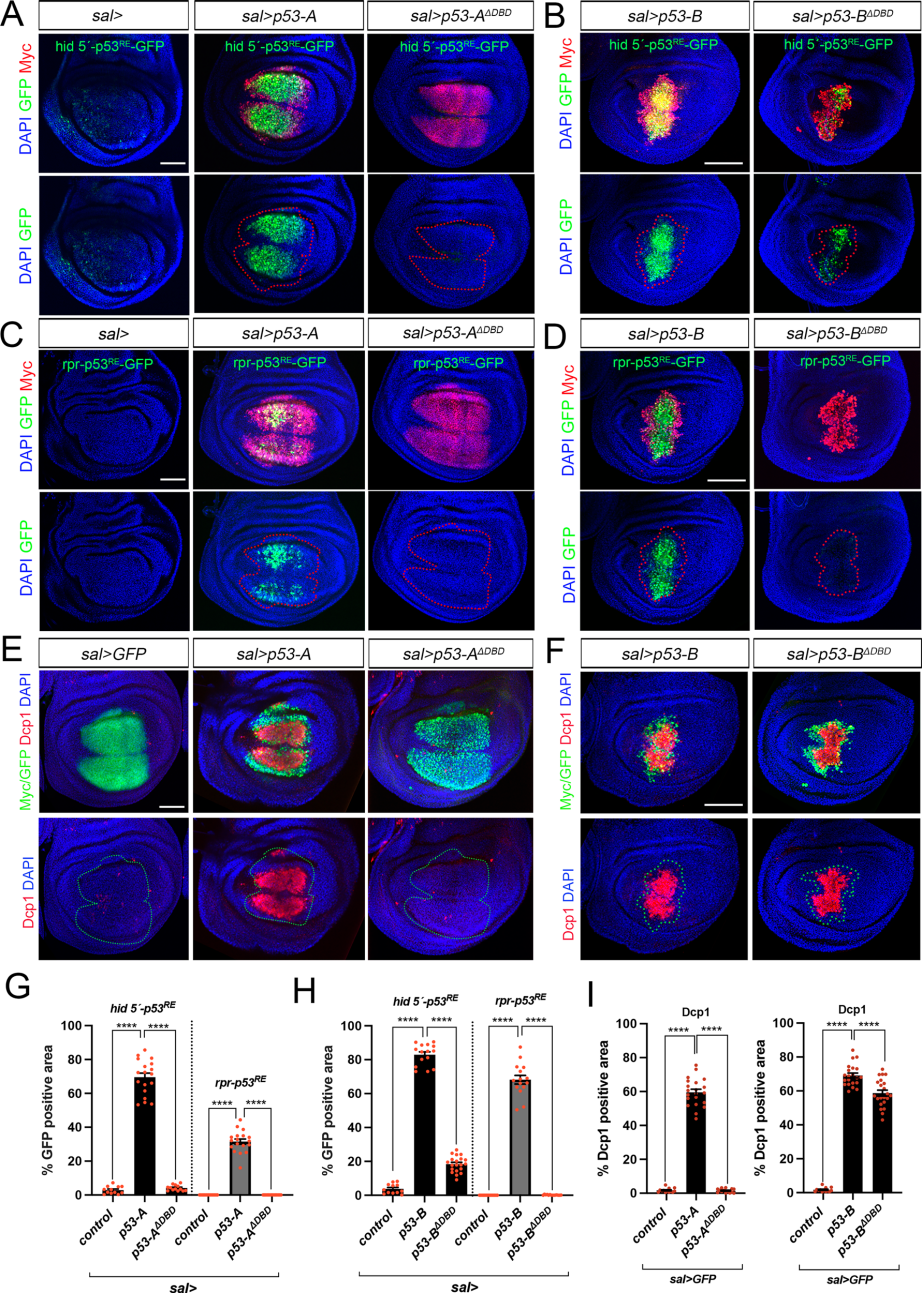
Transcriptional and non-transcriptional roles of p53-A and p53-B isoforms in apoptosis induction. **A–D** Wing imaginal discs expressing the corresponding UAS transgenes under the *sal>* driver. **A**,**B** show the analysis of the activity of the *hid* 5′-p53^RE^-GFP (green) construct, while in (**C**) and (**D**) show the analysis of the *rpr* 5′-p53^RE^-GFP construct. In all images, the Myc (red) and DAPI (blue) channels are also shown. A dotted red line marks the *sal* domain. The scale bar is 50 μm. **E**,**F** Dcp1 staining (red) in wing imaginal discs expressing the indicated UAS transgenes under *sal>* driver. GFP or Myc staining (green), as well as DAPI staining is also shown. A green dotted line marks the *sal* domain. The scale bar is 50 μm. **G**,**H** Quantification of *hid* 5′-p53^RE^-GFP or *rpr-*p53^RE^-GFP positive area in the *sal* domain of wing imaginal discs from the presented genotypes. The data are derived from three independent biological replicates, analyzing more than 13 discs per genotype. Error bars indicate SEM. ****P value < 0.0001 by one-way ANOVA. **I** Quantification of Dcp1 staining in the *sal* domain of wing imaginal discs from the genotypes presented in (**E**) and (**F**). The data are derived from three independent biological replicates, analyzing more than 15 discs per genotype. Error bars indicate SEM. ****P value < 0.0001 by one-way ANOVA.

Our results suggest that the p53 isoforms can induce apoptosis through distinct mechanisms: p53-A and p53-E act as conventional transcription factors regulating the expression of the pro-apoptotic genes, while p53-B can also induce cell death in a transcription-independent manner. To further investigate this hypothesis, we generated new UAS versions of p53-A and p53-B in which the entire DNA binding domain (DBD) was deleted. To facilitate tracking and confirm their expression and nuclear localization, we tagged these versions with Myc (Fig. S3). First, we tested the ability of the p53-A and B isoforms, both with and without their DBDs, to activate the expression of two reporter constructs containing the regulatory regions of the *hid* and *rpr* genes, which include p53 RE (p53^RE^) and to induce apoptosis [18, 43](Fig. 4). In contrast to wild-type p53-A, expression of the p53-A variant lacking the DBD (*p53-A*^*ΔDBD*^) failed to activate the *hid 5´-p53*^*RE*^ and *rpr-p53*^*RE*^ reporters or induce apoptosis (Fig. 4A, C, E, G, I). These results confirm that cell death induction by p53-A depends on its transcriptional activity to regulate the expression of the pro-apoptotic genes. Similarly to p53-A, p53-B without the DBD (p53-B^ΔDBD^) failed to properly activate the *hid 5´-p53*^*RE*^ and *rpr-p53*^*RE*^ reporter genes (Fig. 4B, D, H). However, the robust Dcp-1 immunoreactivity indicated that its apoptotic potential was still strong (Fig. 4F, I). Although p53-B^ΔDBD^ still activates low levels of *hid 5’-p53*^*RE*^, these levels are lower than those induced by wild-type p53-B (Fig. 4B, H). The remaining reporter activation may be due to activated JNK signaling (see below).

Importantly, p53-B^ΔDBD^ can activate the Dronc sensor (Fig. S4A, B), and the cell death triggered by p53-B^ΔDBD^ was completely suppressed by inhibiting Dronc activity (Fig. S4C, D). By contrast, blocking the expression of upstream pro-apoptotic genes did not prevent this apoptosis (Fig. S4C, D). These data further support our hypothesis that p53-B can induce apoptosis by enhancing Dronc activation independently of transcriptional mechanisms.

In order to determine whether p53-B^ΔDBD^ can promote apoptosis through its interaction with the endogenous p53 protein, we expressed *p53-B*^*ΔDBD*^ in a mutant background for a deletion of the entire endogenous p53 gene (*p53*^*5A14*^). We observed that the levels of apoptosis induced by p53-B^ΔDBD^ in this mutant background were similar to those observed in control wild type discs (Fig. S4C, E).

To investigate whether p53-B^ΔDBD^ could promote apoptosis through a non-transcriptional mechanism involving JNK activation, we first tested its ability to activate the JNK pathway transcriptional reporter *TRE-RFP*. Consistent with our previous findings, both p53-A and B robustly activated *TRE-RFP* (Fig. S5A). However, whereas *TRE-RFP* activation by p53-A depended entirely on its DBD, p53-B^ΔDBD^ retained the capacity to induce *TRE-RFP* expression at levels comparable to those of the full-length isoform (Fig. S5A). JNK activation may also be reinforced by a positive feedback loop mediated by the apical caspase Dronc [39, 44]. This further amplifies the JNK pathway downstream of the initial activation of proapoptotic genes. Importantly, JNK activation by p53-B is independent of Dronc, as p53-B still activates TRE in a mutant background for this gene (Fig. S5A) [39]. These results suggest that the residual activation observed with the *hid 5’-p53*^*RE*^ reporter (Fig. 4B, H) may be due to JNK signaling induced by p53-B^ΔDBD^.

Next, we tested whether the apoptosis induced by p53-B^ΔDBD^ depends on JNK activity. To do this, we co-expressed *p53-B*^*ΔDBD*^ and *bsk*^*DN*^ in the *sal* domain. No significant reduction in Dcp1 activation was observed compared to p53-B^ΔDBD^ expression alone (Fig. S5B, C), indicating that apoptosis induced by p53-B^ΔDBD^ proceeds independently of JNK signaling.

Taken together, our findings suggest that p53 isoforms induce cell death through distinct molecular mechanisms. p53-A functions as a canonical transcription factor, regulating the expression of pro-apoptotic genes, such as *rpr* and *hid*. In contrast, p53-B can trigger apoptosis independently of transcriptional programs, primarily relying on the direct activation of the initiator caspase Dronc.

### p53 isoforms can promote senescence-associated tumorigenesis by activating JNK signaling through transcriptional and non-transcriptional mechanisms

Our previous results demonstrated that p53 activation in apoptosis-deficient cells leads to sustained JNK signaling and a senescence-like state, which in turn drives non-autonomous overgrowth of surrounding tissues [35]. To explore the capacity of distinct p53 isoforms to induce a senescence-like state, as well as to assess their potential roles in tumorigenesis, we ectopically expressed the isoforms in the prospective wing region using the *nub-Gal4* driver. These experiments were performed in both apoptosis-competent cells and under different conditions of apoptosis inhibition to define context-dependent effects.

**Fig. 5:**
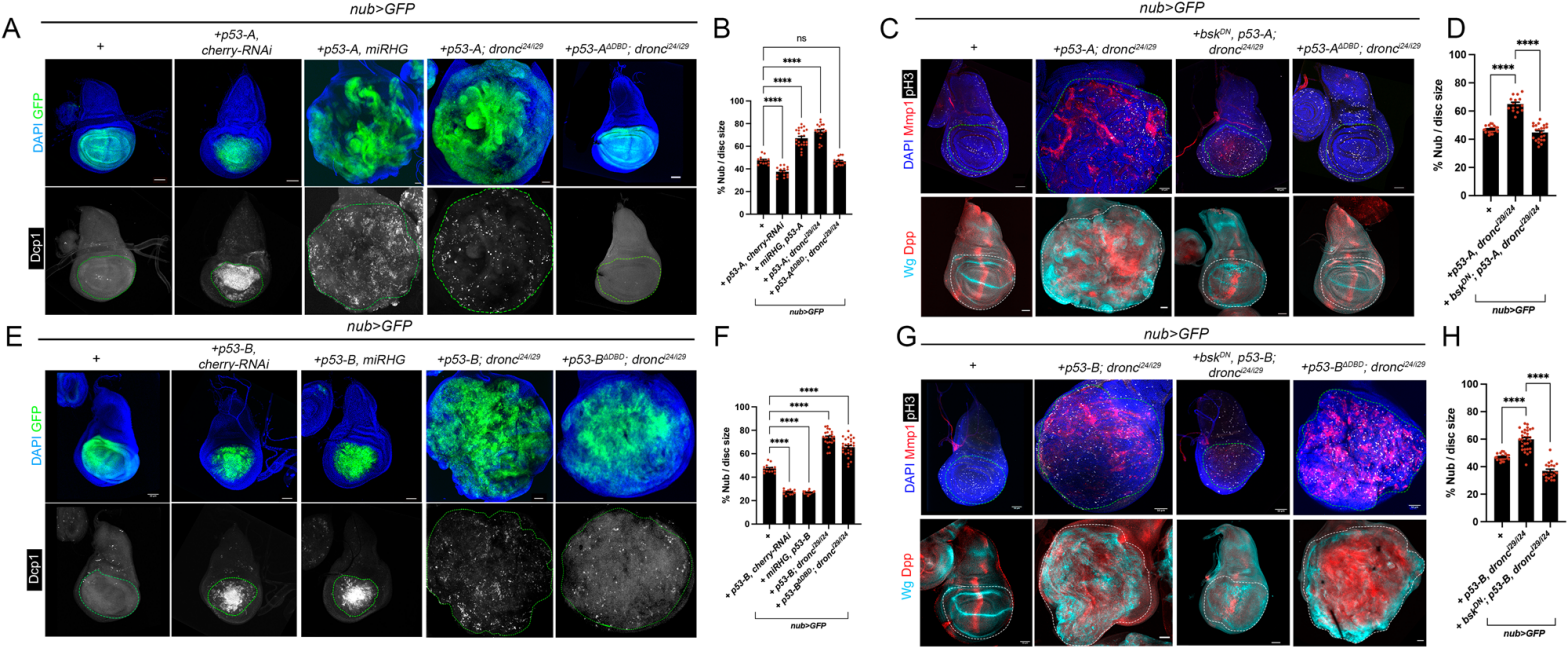
p53 isoforms induced senescence-associated tumorigenesis by activating JNK signaling through transcriptional and non-transcriptional mechanisms. **A**,**C**,**E**,**G** Representative images of wing imaginaldiscs (*n* > 10) expressing the corresponding transgenes with the *nub>* driver. When indicated the over-expression of the different versions of p53-A and p53-B was performed in a *dronc* mutant background. Imaginal discs were stained for GFP (green), DAPI (blue) and Dcp1 (white) in (**A**) and (**E**) or MMP1 (red), pH3 (white), DAPI (blue), Wg (cyan) and Dpp (red) in (**C**) and (**G**). A dotted green line marks the *nub* domain. The scale bar is 50 μm. **B**,**F** Quantification of the tumor overgrowths from the genotypes presented in (**A**) and (**E**) calculated as percentage of the *nub* domain. The data are derived from three independent biological replicates, analyzing more than 15 discs per genotype Error bars indicate SEM. ****P value < 0.0001 and not significant (ns) P value > 0.05 by one-way ANOVA. **D**,**H** Quantification of the tumor overgrowths from the genotypes indicated in (**C**) and (**G**) calculated as percentage of the *nub* domain. The data are derived from three independent biological replicates, analyzing more than 15 discs per genotype. Error bars indicate SEM. ****P value < 0.0001 by one-way ANOVA.

As expected, the expression of *p53-A* in wild type discs induced apoptosis, resulting in a reduction of the *nub* domain. However, in RHG or Dronc deficient cells, p53-A promoted a strong overgrowth of the Nub domain (Fig. 5A, B). In these apoptotic-deficient conditions, p53-A activates the JNK signaling pathway, as visualized by the upregulation of MMP1 expression, along with the induction of the signaling molecules Wg and Dpp (Fig. 5C) [35]. JNK signaling is essential for promoting the overgrowth observed in these mutant conditions, as expression of *bsk*^*DN*^ completely blocks the formation of these tumors and the activation of Wg and Dpp (Fig. 5C, D). Consistent with our previous observations, the expression of the *p53-A* form lacking the DNA binding domain (p53-A^ΔDBD^) in apoptotic-deficient cells fails to induce JNK activation or tumor formation (Fig. 5A–C).

Similar results to those found for p53-A were observed for the p53-E isoform. These included activation of the JNK pathway in apoptotic-deficient cells and formation of overgrowths, both of which were suppressed by the expression of *bsk*^*DN*^ (Fig. S6).

Next, we tested whether the p53-B isoform could also induce the formation of senescent cells and tumor overgrowth under different conditions of apoptotic-deficient cells. Expression of p53-B using the *nub-Gal4* driver in wild-type discs induces cell death and consequently, a reduction of the Nub domain (Fig. 5E, F). As shown previously, knockdown of pro-apoptotic genes neither rescues p53-B-mediated cell death nor induces tumor-like overgrowths (Fig. 5E, F). However, in the absence of Dronc, where apoptosis is strongly suppressed as demonstrated previously, the JNK pathway becomes activated, resulting in the upregulation of Wg and Dpp, which suggests the formation of senescent cells (Fig. 5G). This leads to substantial enlargement of the Nub domain and the development of tumor overgrowths (Fig. 5E–G). As with p53-A and p53-E, these tumor phenotypes depend on JNK activity, as blocking this pathway suppresses the overgrowths (Fig. 5G, H). Remarkably, the formation of these tumor overgrowths does not depend on the transcriptional activity of p53-B, as the expression of *p53-B*^*ΔDBD*^ in a *dronc* mutant background also generates senescent cells, as visualized by MMP1 staining, the upregulation of *wg* and *dpp* expression, and the overgrowth of the *nub* domain (Fig. 5G).

These results suggest that in the absence of apoptosis, all p53 isoforms have tumorigenic potential by activating the JNK pathway and forming senescent cells. However, the mechanism behind JNK activation differs between the different p53 isoforms.

### Analysis of human p53 functions in *Drosophila*

**Fig. 6:**
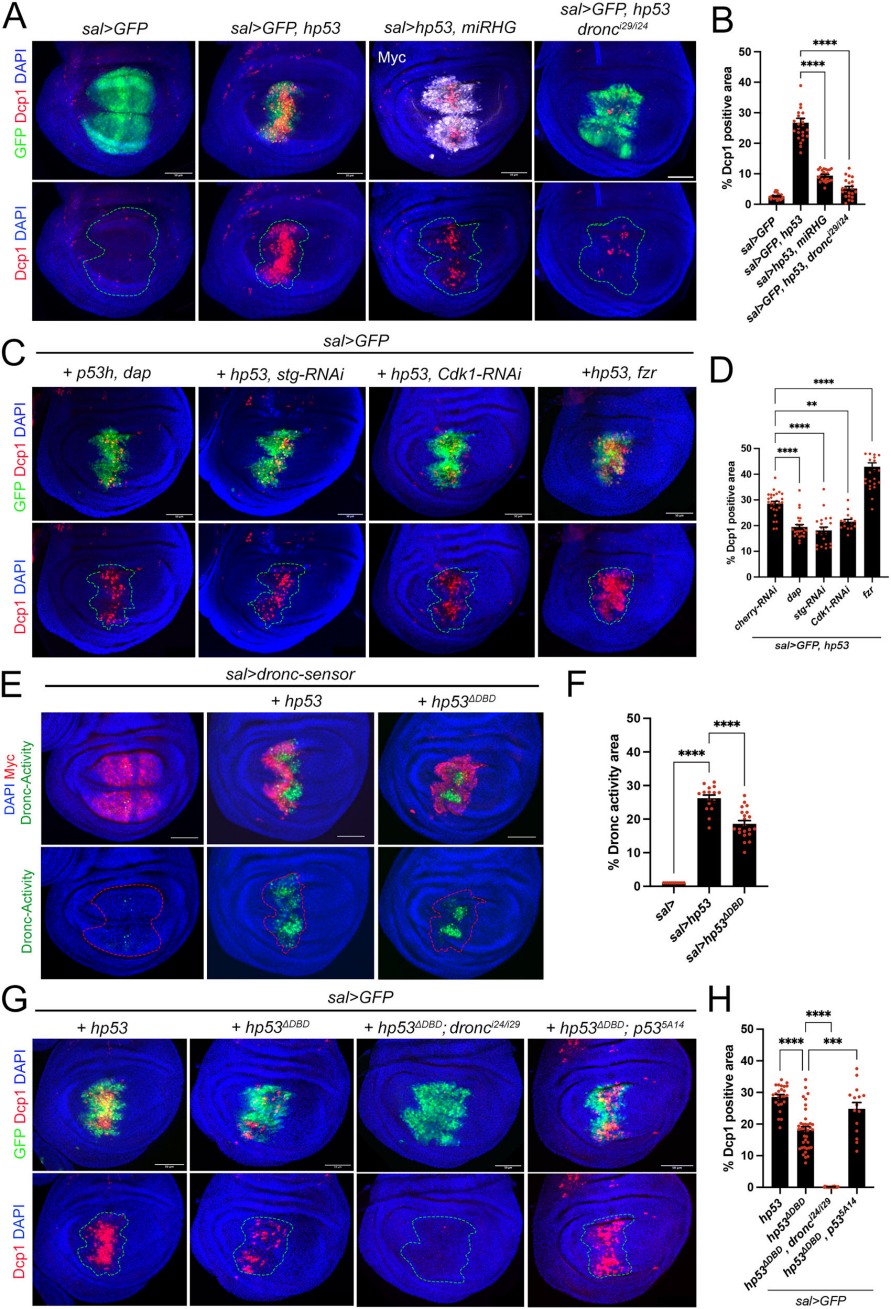
Analysis of apoptotic induction by the expression of *hp53.* **A** Wing imaginal discs expressing the corresponding UAS transgenes under the *sal>*driver. When indicated the expression of the hp53 was performed in a *dronc* mutant background. Imaginal discs were stained for GFP (green), DAPI (blue), Myc (white, only when indicated) and Dcp1 (red). A dotted green line marks the *sal* domain. Scale bar is 50 μm. **B** Quantification of Dcp1 staining in the *sal* domain of wing imaginal discs from the genotypes presented in (**A**). The data are derived from three independent biological replicates, analyzing more than 15 discs per genotype. Error bars indicate SEM. ****P value < 0.0001 by one-way ANOVA. **C** Wing imaginal discs expressing UAS-*hp53* in different conditions of cell cycle arrest stained for GFP (green), DAPI (blue) and Dcp1 (red). **D** Quantification of Dcp1 staining in the *sal* domain of wing imaginal discs from the genotypes presented in (**C**). The data are derived from three independent biological replicates, analyzing more than 15 discs per genotype. Error bars indicate SEM. ****P value < 0.0001 and **P value < 0.001 by one-way ANOVA. **E** Wing imaginal discs expressing the Dronc activity sensor under the *sal>* driver and the corresponding transgenes. The imaginal discs were stained for GFP (green), Myc (red) and DAPI (blue). A dotted red line marks the *sal* domain delimited by Myc staining. The scale bar is 50 μm. **F** Quantification of GFP (Dronc activity) in the *sal* domain of wing imaginal discs from the genotypes presented in (**E**). Error bars indicate SEM. The data are derived from three independent biological replicates, analyzing more than ten discs per genotype. ****P value < 0.0001 by one-way ANOVA. **G** Wing imaginal discs expressing UAS-*hp53*^*ΔDBD*^ with the *sal* > *GFP* driver in a wildtype, *dronc* and *p53* mutant backgrounds stained for GFP (green), DAPI (blue) and Dcp1 (red). A dotted green line marks the *sal* domain. The scale bar is 50 μm. **H** Quantification of Dcp1 staining in the *sal* domain of wing imaginal discs from the genotypes presented in (**G**). The data are derived from three independent biological replicates, analyzing more than 15 discs per genotype. Error bars indicate SEM. ****P value < 0.0001 and ***P value < 0.005 by one-way ANOVA.

Human p53 is required for DNA damage-induced apoptosis [45]. Cdk1 phosphorylates p53 to promote its binding site preference, target selection, and transcriptional response [46–48]. These results suggest that the function of human p53 (hp53) may be modulated by the stage of the cell cycle. However, it remains unclear how the cell cycle influences the apoptotic function of hp53, or whether the interaction between hp53 and Cdk1 is necessary for the induction of apoptosis. In *Drosophila*, we have previously shown that Cdk1 is required for p53-mediated apoptosis [13]. Using this model, we investigated whether human p53-induced apoptosis is similarly regulated by the proliferative state of the cell and whether it depends on Cdk1. Human p53 encodes at least for 12 protein isoforms [14]. TAp53α is the full-length isoform and the most abundant one, containing the entire TAD and the longest C-terminal domain. We generated a transgenic line able to ectopically express TAp53α (hereafter referred to as hp53) under the UAS repeats in *Drosophila*. When expressed in the wing disc cells and the salivary glands, hp53 was predominantly localized in the nuclei, with a small fraction observed in the cytoplasm (Fig. S3). As previously described for the *Drosophila* p53-A and p53-E isoforms, hp53-mediated apoptosis in the wing pouch requires the expression of pro-apoptotic genes and the initiator caspase Dronc (Fig. 6A, B). Consistent with this observation, hp53 expression strongly activates the p53 responsive element (p53^RE^) of the *hid* reporter while weakly activating the *rpr* reporter (Fig. S7A). Next, we explored the impact of cell cycle progression on hp53-induced apoptosis. We found that arresting the cell cycle in G1 by expressing *dap* or in G2 by downregulating *stg* or *Cdk1* significantly reduced hp53-mediated apoptosis when compared with discs in which hp53 was expressed in proliferating cells (Fig. 6C, D). However, this reduction was less pronounced than that observed when the cell cycle was arrested and the *Drosophila p53-A* isoform was overexpressed (compare Fig. 6C, D with Fig. 1C, D). Surprisingly, inducing the endocycle by expressing *fzr*, did not reduce apoptosis, as seen when the *Drosophila p53-A* isoform is expressed, instead it led to an increase in cell death (compare Fig. 6C, D with Fig. 1C, D). As the full-length hp53 isoform shares structural similarities with p53-B [24, 26, 49], which can induce apoptosis in a transcription-independent manner, we generated a human variant form lacking the DNA binding domain (hp53^ΔDBD^) to assess its ability to induce apoptosis. Remarkably, hp53^ΔDBD^ can activate the Dronc sensor and induce apoptosis in a transcription-independent but Dronc-dependent manner (Fig. 6E–H). However, the activation of Dronc and the induction of apoptosis by hp53^ΔDBD^ is much reduced when compared to the full length hp53. To exclude the possibility that hp53^ΔDBD^ interacts with the endogenous *Drosophila* p53 isoforms to trigger the apoptotic pathway, *hp53*^*ΔDBD*^ was expressed in *p53* mutant flies. In this mutant condition, where hp53^ΔDBD^ is the only available p53 isoform, we still observed a strong apoptotic response (Fig. 6G, H). We also tested the ability of both hp53 and hp53^ΔDBD^ to activate the JNK pathway. While both of hp53 versions were equally effective at inducing TRE activity, the expression of *bsk*^*DN*^ did not suppress their ability to induce apoptosis (Fig. S7B, C).

**Fig. 7:**
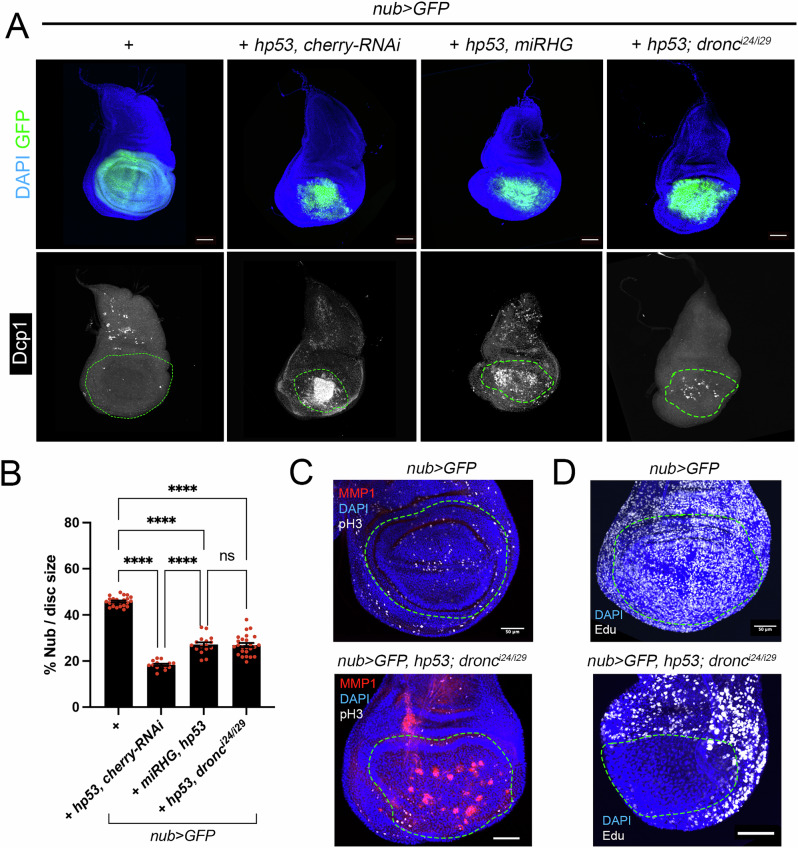
Expression of *hp53* is not able to promote overgrowth in the *nub* domain in apoptotic deficient conditions. **A** Wing imaginal discs expressing *hp53* with the *nub>* driver. When indicated the expression of hp53 was combined with the UAS-*miRHG* or performed in a *dronc* mutant background. Imaginal discs were stained for GFP (green), DAPI (blue) and Dcp1 (white). A dotted green line marks the *nub* domain. The scale bar is 50 μm. **B** Quantification of the tumor overgrowths from the genotypes indicated in (**A**), calculated as a percentage of the *nub* domain. The data are derived from three independent biological replicates, analyzing more than ten discs per genotype. Error bars indicate SEM. ****P value < 0.0001 and not significant (ns) *P* value > 0.05 by one-way ANOVA. **C**,**D** Representative wildtype wing imaginal disc and disc expressing hp53 in a *dronc* mutant background (*n* > 10) stained for GFP (green), DAPI (blue), MMP1 (red) and pH3 (white) in (**C**) or GFP (green), DAPI (blue) and EdU (white). A dotted green line marks the *nub* domain. Scale bar is 50 μm.

Next, we investigated whether the expression of *hp53* in apoptosis-deficient cells lead to tumor overgrowths similar to those observed with *Drosophila* orthologues. We found that hp53 was unable to promote overgrowth in the *nub* domain in either RHG-knockdown or Dronc-deficient cells, although cell death was significatively reduced (Fig. 7A, B). Instead, we observed a strong decrease in the wing area region compared to the wild type control. Importantly, the absence of overgrowth is not due to the inability of hp53 to activate the JNK pathway (Fig. 7C). These results suggest that, unlike the *Drosophila* p53 protein, the human version of p53 induces cell cycle arrest. To confirm this hypothesis, we analyzed cell proliferation markers and a strong reduction in DNA replication was observed as indicated by decreased EdU staining, which could explain the absence of tumor overgrowth (Fig. 7C, D).

Together, these results suggest that the expression of human p53 in *Drosophila* induces apoptosis, which is dependent on the pro-apoptotic genes and is partially influenced by the proliferation state of the cell. Furthermore, hp53 can also trigger apoptosis in a transcription-independent manner. In apoptotic-deficient cells, human p53 activates the JNK pathway; however, this does not lead to tumor overgrowth, likely due to a strong p53-mediated cell cycle arrest. These findings suggest that several functional features of *Drosophila* p53 isoforms are evolutionarily conserved in the human protein.

## Discussion

In this study, we analyzed the apoptotic functions of *Drosophila* p53 isoforms, and the full-length human p53, focusing on their regulation by the cell cycle and their ability to induce senescence-associated overgrowths in apoptotic-deficient cells. Our results suggest that some cellular outcomes directed by p53 depend on the ability of specific isoforms to regulate cell-type-specific transcriptional programs, while others rely on selective protein–protein interactions that are largely independent of p53’s transcriptional activity. These findings provide new insights in our understanding of how p53 operates in vivo and regulates numerous cellular processes.

### Induction of apoptosis

Although p53-A and p53-E promote apoptotic activity through the transcription of *rpr* and *hid*, we discovered that p53-B can induce cell death independently of these pro-apoptotic genes and without relying on its own transcriptional activity. These results are consistent with the cell cycle regulation of p53-A and p53-E by Cdk1 but not of p53-B. Previously, we demonstrated that Cdk1 regulates the binding of p53-A to the p53-RE of *rpr* and *hid* [13]. However, since p53-B can interact with and activate the downstream apical caspase Dronc, it may bypass Cdk1 regulation and induce apoptosis in cell cycle-arrested cells, independently of transcription. It will be interesting to investigate the mechanism behind Dronc activation by p53-B.

Many reports have highlighted the non-canonical roles of human p53 isoforms, which are independent of transcriptional activity, and are particularly important for inducing apoptosis [50–54]. Human p53 can induce apoptosis through multiple transcription-independent mechanisms, primarily by interacting directly with mitochondrial proteins, such as Bcl2 family members and caspases [53–60]. In humans, p53 directly binds to the anti-apoptotic Bcl-2 and Bcl-XL proteins in the mitochondria, thereby displacing and activating pro-apoptotic factors, such as Bax and Bak [54, 61]. This interaction induces mitochondrial outer membrane permeabilization (MOMP), releasing cytochrome c and activating caspase cascades [55]. In addition, p53 can directly activate Bax by catalyzing its translocation to the mitochondria [60], as well as forming a complex with caspase-8 under genotoxic stress (e.g., irradiation) [59], thereby bypassing the need for transcriptional activity. Importantly, this p53 transcription independent apoptosis requires caspase-9 (Dronc orthologue) [59], suggesting that a similar mechanism as the one described for *Drosophila* p53-B could be employed by human p53. Accordingly, when expressed in *Drosophila*, full-length human p53 can induce apoptosis, and part of this effect occurs independently of transcriptional activity, relying instead on Dronc activation.

### Senescence-associated induced tumorigenesis

Post-translational modifications and proteosome degradation ensure that p53 is only transiently activated. However, prolonged cellular stress results in sustained p53 activity, which contributes to the induction of cellular senescence [62]. Although, senescence acts as a tumor-suppressive mechanism by preventing the proliferation of damaged cells, senescent cells can also promote tumor development through the effects of the senescence-associated secretory phenotype (SASP) [63–65]. We have shown that the expression of p53-A in apoptotic-deficient cells results in a strong activation of the JNK pathway and the generation of cells with senescence-associated features [35]. Importantly, these cells secrete signaling molecules, including Upd, Wg and Dpp, which promote the overgrowth of surrounding tissues in a JNK-dependent manner [35]. Notably, p53-A requires its transcriptional activity both to activate JNK signaling and to promote tumorigenesis. In contrast, the p53-B isoform does not require the DBD to activate JNK, induce senescence or promote tissue overgrowth in apoptosis-deficient cells. Consistent with our previous results, p53-B only promotes senescence-associated tumorigenesis when apoptosis is blocked at the level of Dronc. Previous studies have shown that both human and *Drosophila* p53 can stimulate JNK activity independently of their transcriptional function by preventing JNK dephosphorylation and subsequent inactivation [38]. Our results suggest the existence of distinct mechanisms by which p53 isoforms may activate the JNK pathway, since p53-A requires the DBD, whereas p53-B does not.

We also assessed the ability of human p53 to induce senescence-associated tumorigenesis in the absence of apoptosis in *Drosophila*. Unlike fly isoforms, full-length human p53 is unable to sustain tissue overgrowth, as it strongly represses cell cycle progression, mirroring its well-established function in the human context [1, 66]. In contrast to its vertebrate orthologue, *Drosophila* p53 is not required for cell cycle arrest following DNA damage [18–22]. These findings suggest that human p53 retains its cell cycle regulatory function in *Drosophila* effectively blocking cell proliferation and preventing tumor formation.

As p53-B is structurally equivalent to the full-length hp53 (TAp53α), while p53-A is homologous to the amino-terminally truncated isoform of hp53 (Δ40p53α) [24, 26, 49], our results are informative of the molecular mechanisms that regulate the apoptotic and tumor-promoting functions of the different hp53 isoforms. These results highlight the utility of *Drosophila* as a model for studying p53 functions, and provide a platform for investigating human-specific p53 functions that are absent in *Drosophila*.

#### Limitations of the study

Our conclusions are based on overexpression experiments in which different p53 isoforms were expressed in the same tissue under the same conditions. In this controlled experimental setup, the differential functions of p53 isoforms in regulating apoptosis and senescence-associated tumorigenesis are attributed to the distinct protein domains rather than the absence of cell-specific cofactors. However, loss-of-function experiments will be required to analyze the different transcription-dependent and -independent functions of p53 isoforms during development and under stress conditions.

## Material and Methods

### Drosophila strains

The different *Drosophila melanogaster* lines were maintained on standard medium at 25 °C in light/dark cycles of 12 h. The sex of experimental larvae was only considered relevant when selecting for specific mutations that were X-linked. The strains used in this study are: *sal-Gal4* (sal^EPv^-Gal4, FlyBase FBtp0021755), *nub-Gal4* (FlyBase FBtp0080035), UAS-*stg-RNAi* (BDSC 34831), UAS-*Cdk1-RNAi* (BDSC 40950), UAS-*dap* (FlyBase FBgn0010316), UAS-*fzr* (Flybase FBti0064522), UAS-*cherry-RNAi* (BDSC 35785), UAS-*6xMyc:p53-*A [28], UAS-*6xMyc:p53-B* [28], UAS-*6xMyc:p53-E* [28], UAS-*miRHG* [67], *yw hs-flp tub-gal4, UAS-GFP; tub-Gal80 FRT2A/DfH99 FRT2A* (FlyBase FBab0022359), UAS-*Diap1* (FBgn0260635), UAS-*dronc-GFP-TETDG-Myc* (Dronc sensor, this work), *Dronc-HA* [68], *dronc*^*i24*^ and *dronc*^*i29*^ are amorphic alleles (FlyBase FBgn0026404), UAS-*dronc-VC* [69], UAS-*VN-p53-B* (this work), UAS-*VN-p53-B*^*ΔTAD*^ [13], UAS-*p53-A-6xMyc* (86 F) (this work), UAS-*p53-A*^*ΔDBD*^*-6xMyc* (86 F) (this work), UAS-*p53-B-6xMyc* (86 F) (this work), UAS-p53-B^ΔDBD^-6xMyc (86 F) (this work), UAS-*hp53*^*(1-393)*^*-6xMyc* (this work), UAS-*bsk*^*DN*^ (BDSC 6409), UAS-*hep*^*CA*^ (Flybase FBtp0011052), *hid 5*′*-p53*^*RE*^*-GFP* (BDSC 50751), *rpr-p53*^*RE*^*-GFP* [70], *TRE-RFP* [71], UAS-*Mito-GFP* (BDSC 8442) and *p53*^*5A14*^ is a complete deletion of *p53* (BDSC 6815).

To examine the autonomous effects of different p53 isoforms under the various conditions tested, we analyzed only the *sal* domain, using Myc-tagged p53 or GFP expression to define its boundaries. For many of the experiments, we equilibrate the number of UAS transgenes using the UAS-*cherry-RNAi*.

A complete list of the genotypes used for each figure is listed in Table S1.

### Generation of DfH99 mutant cells that overexpressed p53-A and p53-B

Larvae of the following genotypes were heat shocked for 1 h at 37 °C and dissected 48 h after:

*1) yw hs-flp tub-gal4, UAS-GFP; UAS-p53-A:6xMyc/+; tub-Gal80 FRT2A/FRT2A*

*2) yw hs-flp tub-gal4, UAS-GFP; UAS-p53-A:6xMyc/+; tub-Gal80 FRT2A/DfH99 FRT2A*

*3) yw hs-flp tub-gal4, UAS-GFP; UAS-p53-B:6xMyc/+; tub-Gal80 FRT2A/FRT2A*

*4) yw hs-flp tub-gal4, UAS-GFP; UAS-p53-B:6xMyc/+; tub-Gal80 FRT2A/DfH99 FRT2A*

At least 15 clones were recovered from each genotype.

### Imaginal disc staining, acquisition, and processing

Third instar larvae were dissected in PBS and fixed in a solution of 4% paraformaldehyde, 0.1% deoxycholate, and 0.1% Triton X-100 in PBS for 28 min at room temperature. The samples were then blocked in PBS, containing 1% BSA, and 0.3% Triton X-100 for 1 h, after which they were incubated with the primary antibody overnight at 4 °C, washed four times in washing buffer (PBS 0.3% Triton X-100) and incubated with the appropriate fluorescent secondary antibodies for 1.5 h at room temperature in the dark. The samples were then washed and mounted in Vectashield (Cat# H-1000 RRID: AB_2336790) for confocal analysis.

Antibodies used: pH3 (Rabbit, 1:1000, MerckMillipore #06-570), pH3 (mouse, 1:1000, Cell signal technology #9706), Dcp1 (Rabbit, 1:200, Cell signal technology #9578), Myc (mouse, 1:200, DHSB #9E 10), MMP1 (mouse, 1:50, DHSB #5H7B11, #3A6B4, #3B8D), Wg (mouse, 1:50, DHSB #4D4), p53 (mouse, 1:50 DHSB #25F4) and Dpp (rabbit, 1:200) [72].

All confocal images were obtained using an LSM710 vertical confocal microscope and an Olympus SpinSR10. Multiple Z-stacks were obtained for each imaginal disc. Image processing and analysis were performed using Fiji (https://fji.sc) and Adobe Photoshop software.

To quantify Dcp1 and GFP a Z-maximal intensity projection was generated for each image and a high-intensity threshold was adjusted for each image. Then, we calculated the percentage of staining covered in the region of interest.

For Venus intensity levels, a Z-maximum projection was generated for each image and the mean intensity levels were obtained for Venus and p53 staining and subtracted the background intensity.

For TRE-RFP intensity levels, a Z-stack maximum projection was generated for each image and the mean intensity levels for RFP were obtained and then subtracted the background intensity.

All discs were dissected from multiple biological replicates and the number of discs analyzed in each experiment is provided in the figure legends. Statistical analysis was performed using GraphPad Prism software (https://www.graphpad.com). The specific statistical test and the n value used in each analysis are noted in the corresponding figure.

### EdU treatment

For EdU staining, the dissected discs were cultured in 1 ml of EdU labeling solution for 30 min at room temperature and subsequently fixed in 4% paraformaldehyde for 30 min at room temperature. EdU detection was performed according to the manufacturer’s instructions (Click-iT EdU Alexa Fluor Imaging Kit, Thermo Fisher Scientific).

### BiFC assay

We used a pUAST attB with the N-terminal (VN: 1-173) and C-terminal (VC: 155–238) moieties of Venus cloned into the Xho1 and Xba1 restriction sites. The coding region of p53-B was PCR amplified from UAS-*p53-B* flies. The insert was cloned into the pUAST attB VN version with Xho1-Xba1 sites including a five amino acids linker region. N- terminal deletions of p53-B were cloned in a similar manner. To ensure similar expression levels, all UAS constructs were inserted into the same attP site (86Fb).

UAS-*VN-p53-B* forward:

CAGTCTCGAGGGCGGCTCAGGCGGCATGAGTCTTCACAAGTCCGCGTCGTTTAG (Xho1+Linker)

UAS-*VN-p53-B* reverse:

CAGTTCTAGATCATGGCAGCTCGTAGGCACG (Xba1)

### Generation of the UAS-p53 lines

UAS- *p53-A-6xMy*c:

The coding region of p53-A was PCR amplified from the GH11591 clone (BDGP) with the following primers with EcoR1 flanking sites:

p53-A Forward: CAGTGAATTCATGTATATATCACAGCCAATGTCG (EcoR1)

p53-A Reverse: CAGTGAATTCTGGCAGCTCGTAGGCACGTTTC (EcoR1)

The insert was cloned into the pUAST-attB-6XMyc version at the EcoR1 site.

UAS-*p53-A*^*ΔDBD*^-*6xMyc*:

To delete the DBD (85–276aa) of p53-A, we performed PCR amplification of the N- and C- terminus of p53-A using the following primers and inserted a BglII site for subsequent ligation between the two fragments and the pUAST-attB-6XMyc vector. When ligated, the N and C-terminus of p53 maintain the ORF.

p53-A-N-terminal Forward: CAGTGAATTCATGTATATATCACAGCCAATGTCG (EcoR1)

p53-A-N-terminal Reverse: CAGTAGATCTCTAGCTTGGGCAGCGTGTTCGCC (BglII)

p53-A-C-terminal Forward: CAGTAGATCTATAGCAAGAAGCGCAAGTCCGTGCC (BglII)

p53-A-C-terminal Reverse: CAGTGAATTCTGGCAGCTCGTAGGCACGTTTC (EcoR1)

UAS-*p53-B-6xMyc*:

The coding region of p53-B was PCR amplified with the following primers with EcoR1 flanking sites:

p53-B Forward: CAGTGAATTCATGAGTCTTCACAAGTCCGC

p53-B Reverse: CAGTGAATTCTGGCAGCTAGGCACGTTTC

UAS-*p53-B*^*ΔDBD*^-*6xMyc*:

To delete the DBD (195–386aa) of p53-B we PCR amplified the N- and C- terminus of p53-B with the following primers and inserted a BglII site for subsequent ligation between the two fragments and the pUAST-attB-6XMyc vector. When ligated, the N and C-terminus of p53 maintain the ORF.

p53-B-N-terminal forward: CAGTGAATTCATGAGTCTTCACAAGTCCGC (EcoR1)

p53-B-N-terminal reverse: CAGTAGATCTCTAGCTTGGGCAGCGTGTTCGCC (BglII)

p53-B-C-terminal forward: CAGTAGATCTATAGCAAGAAGCGCAAGTCCGTGCC (BglII)

p53-B-C-terminal reverse: CAGTGAATTCTGGCAGCTCGTAGGCACGTTTC (EcoR1)

UAS-*hp53-6xMyc*:

The coding region of the full length human p53 was PCR amplified from the pGEX hp53 (1-393) Addgene 2486 with the following primers with EcoR1 flanking sites:

hp53 Forward: CAGTGAATTCATGGAGGAGCCGCAGTCAG (EcoR1)

hp53 Reverse: CAGTGAATTCGTCTGAGTCAGGCCCTTCTGTC (EcoR1)

UAS-*hp53*^*ΔDBD*^-*6xMyc*:

To delete the DBD (99–292aa) of hp53 we PCR amplified the N- and C- terminus of hp53 from the pGEX hp53 with the following primers and inserted a AvrII site for subsequent ligation between the two fragments and the pUAST-attB-6XMyc vector. When ligated, the N and C-terminus of hp53 maintain the ORF.

hp53-N-terminal forward: CAGTGAATTCATGGAGGAGCCGCAGTCAG (EcoR1)

hp53-N-terminal reverse CAGTCCTAGGAGGGACAGAAGATGACAGGGG (AvrII)

hp53-C-terminal forward: CAGTCCTAGGGGGGAGCCTCACCACGAGCTG (AvrII)

hp53-C-terminal reverse: CAGTGAATTCGTCTGAGTCAGGCCCTTCTGTC (EcoR1)

To ensure similar expression levels, all UAS constructs were inserted into the same attP sites (51D and 86Fb).

### CoInmunoprecipitation

One hundred imaginal discs from third instar *Drosophila* larvae were dissected in ice-cold phosphate-buffered saline (PBS). Tissues were homogenized in ice-cold RIPA buffer (10 mM Tris-HCl pH 7.6, 1 mM EDTA, 0.1% SDS, 0.1% sodium deoxycholate, 1% Triton X-100) supplemented with protease inhibitors and PMSF. The homogenate was incubated on ice for 30 min with occasional vortexing.

Lysates were clarified by centrifugation at 14,000 X *g* for 20 min at 4 °C. The supernatant was collected as the protein lysate. 400 µL of TBST-EDTA containing protease inhibitors was added to 150 µL of lysate.

Magnetic beads conjugated to anti-Myc (ChromoTek AB_2631370) or anti-HA (ThermoFisher Cat#88836) antibodies were pre-washed with RIPA buffer using a magnetic rack. Fifteen microliters of these antibody-coupled beads were added to the pre-cleared lysates, followed by incubation at 4 °C for 2 h with gentle rotation.

Beads were separated using a magnetic rack, and the unbound lysate was transferred to a fresh tube. The beads were washed 5 times with ice-cold RIPA buffer to remove non-specific interactions. Each wash consisted of adding buffer, gently inverting or mixing, then separating beads with a magnet and discarding the supernatant. To elute bound proteins, SDS-PAGE sample buffer was added to the beads, and samples were heated at 95 °C for 5 min. Eluates were analyzed by SDS-PAGE and western blotting.

Protein samples were resolved on precast 4–10% gradient gels using MES running buffer, and electrophoresis was carried out at 120 V for 45–90 min until the dye front reached the bottom of the gel. Proteins were then transferred to a membrane using semi-dry transfer at 25 V for 30 min. Membranes were blocked in 5% non-fat dry milk in TBST (TBS + 0.1% Tween-20) for 1 h at room temperature. Primary antibody (anti-Myc) was diluted in blocking buffer and incubated with the membrane for 2 h at room temperature. Membranes were washed three times for 10 min each in TBST. Secondary antibody (HRP-conjugated) was diluted in blocking buffer and incubated for 1 h at room temperature, followed by three additional washes in TBST. Detection was performed using chemiluminescent substrates. Uncropped western blots are provided in Supplementary Material.

### Generation of the UAS-dronc-GFP-TETDG-Myc Construct

A wild-type *dronc* cDNA was synthesized (GeneWizz) and fused in-frame to the Suntag and HA tag peptides at the C-terminal end. To facilitate downstream cloning, additional restriction sites were introduced at both the 5ʹ and 3ʹ ends of the construct, as well as upstream of the tag peptide. The full-length construct was initially subcloned into the pUC57 vector as a *NotI-KpnI* fragment.

Subsequently, the vector was digested with *SmaI* and *NheI*, resulting in the removal of the C-terminal Suntag-HA tagging from the wild-type *dronc* sequence. A modified version of GFP, containing a Myc tag at its C-terminal end, was generated by PCR using the primers listed below. This GFP variant includes a TETDG caspase cleavage site, which, upon Dronc-dependent cleavage, restores GFP to a conformational state compatible with fluorescence emission. The template for the GFP-Myc sequence was described previously [68]. The GFP-Myc PCR product was subsequently cloned in-frame at the C-terminal end of wild-type Dronc as a *SmaI-NheI* fragment.

Primers used for GFP-Myc amplification:

- Forward primer: 5ʹ GCTTTAATAAGAAACTCTACTTCAATcccgggtttttcaacgaagggggcATGATCAAGATCGCCACCAGGAAGTACC 3ʹ
- Reverse primer: 5ʹ GATAAAATGTCCAGTGGCGGCAAGCTAGCttacaggtcctcctcgctgatcagcttctgctcGTTAGGCAGGTTGTCCACCCTCATCAGG 3ʹ

The complete construct was then subcloned as a *NotI-XhoI* fragment into a UAS-attB *w+* vector previously linearized with *NotI-PspXI*.

### Figure preparation

Images were analyzed with Fiji and cartoons were created with BioRender.com. Figures were prepared using Adobe Photoshop.

## Supplementary information

Figure S1

Figure S2

Figure S3

Figure S4

Figure S5

Figure S6

Figure S7

Sup. Figure legends

Suplementary Table 1

Uncropped original western blot used in Figure 3H

## Data Availability

All data and materials used in the article are available upon request.

## References

[1] Vousden KH, Lane DP. p53 in health and disease. Nat Rev Mol Cell Biol. 2007;8:275–83. 17380161 10.1038/nrm2147

[2] Hafner A, Bulyk ML, Jambhekar A, Lahav G. The multiple mechanisms that regulate p53 activity and cell fate. Nat Rev Mol Cell Biol. 2019;20:199–210. 30824861 10.1038/s41580-019-0110-x

[3] Muller PA, Vousden KH. p53 mutations in cancer. Nat Cell Biol. 2013;15:2–8. 23263379 10.1038/ncb2641

[4] Kennedy MC, Lowe SW. Mutant p53: it’s not all one and the same. Cell Death Differ. 2022;29:983–7. 35361963 10.1038/s41418-022-00989-y PMC9090915

[5] Dittmer D, Pati S, Zambetti G, Chu S, Teresky AK, Moore M, et al. Gain of function mutations in p53. Nat Genet. 1993;4:42–6. 8099841 10.1038/ng0593-42

[6] Beckerman R, Prives C. Transcriptional regulation by p53. Cold Spring Harb Perspect Biol. 2010;2:a000935. 20679336 10.1101/cshperspect.a000935 PMC2908772

[7] Liu Y, Tavana O, Gu W. p53 modifications: exquisite decorations of the powerful guardian. J Mol Cell Biol. 2019;11:564–77. 31282934 10.1093/jmcb/mjz060 PMC6736412

[8] Murray-Zmijewski F, Slee EA, Lu X. A complex barcode underlies the heterogeneous response of p53 to stress. Nat Rev Mol Cell Biol. 2008;9:702–12. 18719709 10.1038/nrm2451

[9] Tatavosian R, Donovan MG, Galbraith MD, Duc HN, Szwarc MM, Joshi MU, et al. Cell differentiation modifies the p53 transcriptional program through a combination of gene silencing and constitutive transactivation. Cell Death Differ. 2023;30:952–65. 36681780 10.1038/s41418-023-01113-4 PMC10070495

[10] Minter LM, Dickinson ES, Naber SP, Jerry DJ. Epithelial cell cycling predicts p53 responsiveness to gamma-irradiation during post-natal mammary gland development. Development. 2002;129:2997–3008. 12050146 10.1242/dev.129.12.2997

[11] Kurtz P, Jones AE, Tiwari B, Link N, Wylie A, Tracy C, et al. Drosophila p53 directs nonapoptotic programs in postmitotic tissue. Mol Biol Cell. 2019;30:1339–51. 30892991 10.1091/mbc.E18-12-0791 PMC6724604

[12] MacCallum DE, Hupp TR, Midgley CA, Stuart D, Campbell SJ, Harper A, et al. The p53 response to ionising radiation in adult and developing murine tissues. Oncogene. 1996;13:2575–87. 9000131

[13] Ruiz-Losada M, Gonzalez R, Peropadre A, Gil-Galvez A, Tena JJ, Baonza A, et al. Coordination between cell proliferation and apoptosis after DNA damage in Drosophila. Cell Death Differ. 2022;29:832–45. 34824391 10.1038/s41418-021-00898-6 PMC8989919

[14] Marcel V, Dichtel-Danjoy ML, Sagne C, Hafsi H, Ma D, Ortiz-Cuaran S, et al. Biological functions of p53 isoforms through evolution: lessons from animal and cellular models. Cell Death Differ. 2011;18:1815–24. 21941372 10.1038/cdd.2011.120 PMC3214904

[15] Khoury MP, Bourdon JC. The isoforms of the p53 protein. Cold Spring Harb Perspect Biol. 2010;2:a000927. 20300206 10.1101/cshperspect.a000927 PMC2829963

[16] Bourdon JC. p53 and its isoforms in cancer. Br J Cancer. 2007;97:277–82. 17637683 10.1038/sj.bjc.6603886 PMC2360320

[17] Guo Y, Wu H, Wiesmuller L, Chen M. Canonical and non-canonical functions of p53 isoforms: potentiating the complexity of tumor development and therapy resistance. Cell Death Dis. 2024;15:412. 38866752 10.1038/s41419-024-06783-7 PMC11169513

[18] Brodsky MH, Nordstrom W, Tsang G, Kwan E, Rubin GM, Abrams JM. Drosophila p53 binds a damage response element at the reaper locus. Cell. 2000;101:103–13. 10778860 10.1016/S0092-8674(00)80627-3

[19] Ollmann M, Young LM, Di Como CJ, Karim F, Belvin M, Robertson S, et al. Drosophila p53 is a structural and functional homolog of the tumor suppressor p53. Cell. 2000;101:91–101. 10778859 10.1016/S0092-8674(00)80626-1

[20] Lee JH, Lee E, Park J, Kim E, Kim J, Chung J. In vivo p53 function is indispensable for DNA damage-induced apoptotic signaling in Drosophila. FEBS Lett. 2003;550:5–10. 12935877 10.1016/s0014-5793(03)00771-3

[21] Sogame N, Kim M, Abrams JM. Drosophila p53 preserves genomic stability by regulating cell death. Proc Natl Acad Sci USA. 2003;100:4696–701. 12672954 10.1073/pnas.0736384100 PMC153618

[22] Jin S, Martinek S, Joo WS, Wortman JR, Mirkovic N, Sali A, et al. Identification and characterization of a p53 homologue in Drosophila melanogaster. Proc Natl Acad Sci USA. 2000;97:7301–6. 10860994 10.1073/pnas.97.13.7301 PMC16540

[23] Baonza A, Tur-Gracia S, Perez-Aguilera M, Estella C. Regulation and coordination of the different DNA damage responses in Drosophila. Front Cell Dev Biol. 2022;10:993257. 36147740 10.3389/fcell.2022.993257 PMC9486394

[24] Ingaramo MC, Sanchez JA, Dekanty A. Regulation and function of p53: a perspective from Drosophila studies. Mech Dev. 2018;154:82–90. 29800619 10.1016/j.mod.2018.05.007

[25] D’Brot A, Kurtz P, Regan E, Jakubowski B, Abrams JM. A platform for interrogating cancer-associated p53 alleles. Oncogene. 2017;36:292. 27991923 10.1038/onc.2016.190

[26] Joruiz SM, Bourdon JC p53 isoforms: key regulators of the cell fate decision. Cold Spring Harb Perspect Med. 2016;6:a026039. 10.1101/cshperspect.a026039 PMC4968168 26801896

[27] Zhang B, Mehrotra S, Ng WL, Calvi BR. Low levels of p53 protein and chromatin silencing of p53 target genes repress apoptosis in Drosophila endocycling cells. PLoS Genet. 2014;10:e1004581. 25211335 10.1371/journal.pgen.1004581 PMC4161308

[28] Zhang B, Rotelli M, Dixon M, Calvi BR. The function of Drosophila p53 isoforms in apoptosis. Cell Death Differ. 2015;22:2058–67. 25882045 10.1038/cdd.2015.40 PMC4816103

[29] Dichtel-Danjoy ML, Ma D, Dourlen P, Chatelain G, Napoletano F, Robin M, et al. Drosophila p53 isoforms differentially regulate apoptosis and apoptosis-induced proliferation. Cell Death Differ. 2013;20:108–16. 22898807 10.1038/cdd.2012.100 PMC3524635

[30] Robin M, Issa AR, Santos CC, Napoletano F, Petitgas C, Chatelain G, et al. Drosophila p53 integrates the antagonism between autophagy and apoptosis in response to stress. Autophagy. 2019;15:771–84. 30563404 10.1080/15548627.2018.1558001 PMC6526837

[31] Park JH, Nguyen TTN, Lee EM, Castro-Aceituno V, Wagle R, Lee KS, et al. Role of p53 isoforms in the DNA damage response during Drosophila oogenesis. Sci Rep. 2019;9:11473. 31391501 10.1038/s41598-019-47913-y PMC6685966

[32] Chakravarti A, Thirimanne HN, Brown S, Calvi BR Drosophila p53 isoforms have overlapping and distinct functions in germline genome integrity and oocyte quality control. Elife. 2022;11:e613389. 10.7554/eLife.61389 PMC8758136 35023826

[33] Wylie A, Jones AE, Das S, Lu WJ, Abrams JM. Distinct p53 isoforms code for opposing transcriptional outcomes. Dev Cell. 2022;57:1833–46 e6. 35820415 10.1016/j.devcel.2022.06.015 PMC9378576

[34] Napoletano F, Gibert B, Yacobi-Sharon K, Vincent S, Favrot C, Mehlen P, et al. p53-dependent programmed necrosis controls germ cell homeostasis during spermatogenesis. PLoS Genet. 2017;13:e1007024. 28945745 10.1371/journal.pgen.1007024 PMC5629030

[35] Garcia-Arias JM, Pinal N, Cristobal-Vargas S, Estella C, Morata G. Lack of apoptosis leads to cellular senescence and tumorigenesis in Drosophila epithelial cells. Cell Death Discov. 2023;9:281. 37532716 10.1038/s41420-023-01583-y PMC10397273

[36] Edgar BA, Zielke N, Gutierrez C. Endocycles: a recurrent evolutionary innovation for post-mitotic cell growth. Nat Rev Mol Cell Biol. 2014;15:197–210. 24556841 10.1038/nrm3756

[37] White K, Grether ME, Abrams JM, Young L, Farrell K, Steller H. Genetic control of programmed cell death in Drosophila. Science. 1994;264:677–83. 8171319 10.1126/science.8171319

[38] McEwen DG, Peifer M. Puckered, a Drosophila MAPK phosphatase, ensures cell viability by antagonizing JNK-induced apoptosis. Development. 2005;132:3935–46. 16079158 10.1242/dev.01949

[39] Shlevkov E, Morata G. A dp53/JNK-dependant feedback amplification loop is essential for the apoptotic response to stress in Drosophila. Cell Death Differ. 2012;19:451–60. 21886179 10.1038/cdd.2011.113 PMC3278728

[40] Luo X, Puig O, Hyun J, Bohmann D, Jasper H. Foxo and Fos regulate the decision between cell death and survival in response to UV irradiation. EMBO J. 2007;26:380–90. 17183370 10.1038/sj.emboj.7601484 PMC1783446

[41] Wang SL, Hawkins CJ, Yoo SJ, Muller HA, Hay BA. The Drosophila caspase inhibitor DIAP1 is essential for cell survival and is negatively regulated by HID. Cell. 1999;98:453–63. 10481910 10.1016/s0092-8674(00)81974-1

[42] Hudry B, Viala S, Graba Y, Merabet S. Visualization of protein interactions in living Drosophila embryos by the bimolecular fluorescence complementation assay. BMC Biol. 2011;9:5. 21276241 10.1186/1741-7007-9-5 PMC3041725

[43] Tanaka-Matakatsu M, Xu J, Cheng L, Du W. Regulation of apoptosis of rbf mutant cells during Drosophila development. Dev Biol. 2009;326:347–56. 19100727 10.1016/j.ydbio.2008.11.035 PMC2634822

[44] Wells BS, Yoshida E, Johnston LA. Compensatory proliferation in Drosophila imaginal discs requires dronc-dependent p53 activity. Curr Biol. 2006;16:1606–15. 16920621 10.1016/j.cub2006.07.046 PMC1764442

[45] Vousden KH, Prives C. Blinded by the light: the growing complexity of p53. Cell. 2009;137:413–31. 19410540 10.1016/j.cell.2009.04.037

[46] Bischoff JR, Friedman PN, Marshak DR, Prives C, Beach D. Human p53 is phosphorylated by p60-cdc2 and cyclin B-cdc2. Proc Natl Acad Sci USA. 1990;87:4766–70. 2141171 10.1073/pnas.87.12.4766 PMC54198

[47] Blaydes JP, Luciani MG, Pospisilova S, Ball HM, Vojtesek B, Hupp TR. Stoichiometric phosphorylation of human p53 at Ser315 stimulates p53-dependent transcription. J Biol Chem. 2001;276:4699–708. 11078726 10.1074/jbc.M003485200

[48] Wang Y, Prives C. Increased and altered DNA binding of human p53 by S and G2/M but not G1 cyclin-dependent kinases. Nature. 1995;376:88–91. 7596441 10.1038/376088a0

[49] Bourdon JC, Fernandes K, Murray-Zmijewski F, Liu G, Diot A, Xirodimas DP, et al. p53 isoforms can regulate p53 transcriptional activity. Genes Dev. 2005;19:2122–37. 16131611 10.1101/gad.1339905 PMC1221884

[50] Bissonnette N, Hunting WasylykB. DJ. The apoptotic and transcriptional transactivation activities of p53 can be dissociated. Biochem Cell Biol. 1997;75:351–8. 9493957

[51] Caelles C, Helmberg A, Karin M. p53-dependent apoptosis in the absence of transcriptional activation of p53-target genes. Nature. 1994;370:220–3. 8028670 10.1038/370220a0

[52] Haupt Y, Rowan S, Shaulian E, Vousden KH, Oren M. Induction of apoptosis in HeLa cells by trans-activation-deficient p53. Genes Dev. 1995;9:2170–83. 7657168 10.1101/gad.9.17.2170

[53] Marchenko ND, Zaika A, Moll UM. Death signal-induced localization of p53 protein to mitochondria. A potential role in apoptotic signaling. J Biol Chem. 2000;275:16202–12. 10821866 10.1074/jbc.275.21.16202

[54] Mihara M, Erster S, Zaika A, Petrenko O, Chittenden T, Pancoska P, et al. p53 has a direct apoptogenic role at the mitochondria. Mol Cell. 2003;11:577–90. 12667443 10.1016/s1097-2765(03)00050-9

[55] Speidel D. Transcription-independent p53 apoptosis: an alternative route to death. Trends Cell Biol. 2010;20:14–24. 19879762 10.1016/j.tcb.2009.10.002

[56] Schuler M, Bossy-Wetzel E, Goldstein JC, Fitzgerald P, Green DR. p53 induces apoptosis by caspase activation through mitochondrial cytochrome c release. J Biol Chem. 2000;275:7337–42. 10702305 10.1074/jbc.275.10.7337

[57] Baptiste-Okoh N, Barsotti AM, Prives C. A role for caspase 2 and PIDD in the process of p53-mediated apoptosis. Proc Natl Acad Sci USA. 2008;105:1937–42. 18238895 10.1073/pnas.0711800105 PMC2538861

[58] Frank AK, Pietsch EC, Dumont P, Tao J, Murphy ME. Wild-type and mutant p53 proteins interact with mitochondrial caspase-3. Cancer Biol Ther. 2011;11:740–5. 21307660 10.4161/cbt.11.8.14906 PMC3100564

[59] Ding HF, Lin YL, McGill G, Juo P, Zhu H, Blenis J, et al. Essential role for caspase-8 in transcription-independent apoptosis triggered by p53. J Biol Chem. 2000;275:38905–11. 10988287 10.1074/jbc.M004714200

[60] Chipuk JE, Kuwana T, Bouchier-Hayes L, Droin NM, Newmeyer DD, Schuler M, et al. Direct activation of Bax by p53 mediates mitochondrial membrane permeabilization and apoptosis. Science. 2004;303:1010–4. 14963330 10.1126/science.1092734

[61] Wei H, Qu L, Dai S, Li Y, Wang H, Feng Y, et al. Structural insight into the molecular mechanism of p53-mediated mitochondrial apoptosis. Nat Commun. 2021;12:2280. 33863900 10.1038/s41467-021-22655-6 PMC8052441

[62] Mijit M, Caracciolo V, Melillo A, Amicarelli F, Giordano A Role of p53 in the regulation of cellular senescence. Biomolecules. 2020;10:420. 10.3390/biom10030420 PMC7175209 32182711

[63] Collado M, Blasco MA, Serrano M. Cellular senescence in cancer and aging. Cell. 2007;130:223–33. 17662938 10.1016/j.cell.2007.07.003

[64] Sheekey E, Narita M. p53 in senescence - it’s a marathon, not a sprint. FEBS J. 2023;290:1212–20. 34921507 10.1111/febs.16325

[65] Qian Y, Chen X. Senescence regulation by the p53 protein family. Methods Mol Biol. 2013;965:37–61. 23296650 10.1007/978-1-62703-239-1_3 PMC3784259

[66] Kastan MB, Onyekwere O, Sidransky D, Vogelstein B, Craig RW. Participation of p53 protein in the cellular response to DNA damage. Cancer Res. 1991;51:6304–11. 1933891

[67] Siegrist SE, Haque NS, Chen CH, Hay BA, Hariharan IK. Inactivation of both Foxo and reaper promotes long-term adult neurogenesis in Drosophila. Curr Biol. 2010;20:643–8. 20346676 10.1016/j.cub.2010.01.060 PMC2862284

[68] Arthurton L, Nahotko DA, Alonso J, Wendler F, Baena-Lopez LA. Non-apoptotic caspase activation preserves Drosophila intestinal progenitor cells in quiescence. EMBO Rep. 2020;21:e48892. 33135280 10.15252/embr.201948892 PMC7726796

[69] Wendler F, Park S, Hill C, Galasso A, Chang KR, Awan I, et al. A LexAop > UAS > QUAS trimeric plasmid to generate inducible and interconvertible Drosophila overexpression transgenes. Sci Rep. 2022;12:3835. 35264662 10.1038/s41598-022-07852-7 PMC8907290

[70] Lu WJ, Chapo J, Roig I, Abrams JM. Meiotic recombination provokes functional activation of the p53 regulatory network. Science. 2010;328:1278–81. 20522776 10.1126/science.1185640 PMC2917750

[71] Chatterjee N, Bohmann DA. versatile PhiC31 based reporter system for measuring AP-1 and Nrf2 signaling in Drosophila and in tissue culture. PLoS One. 2012;7:e34063. 22509270 10.1371/journal.pone.0034063 PMC3324472

[72] Akiyama T, Gibson MC. Decapentaplegic and growth control in the developing Drosophila wing. Nature. 2015;527:375–8. 26550824 10.1038/nature15730

