# Supplementary figures and images for: Transcription-dependent and -independent functions of Drosophila p53 isoforms in the induction of apoptosis and senescence-associated tumorigenesis
Source: Cell Death Dis. 2026 Mar 25;17(1):367. doi: 10.1038/s41419-026-08571-x (PMC13039399; doi:10.1038/s41419-026-08571-x)

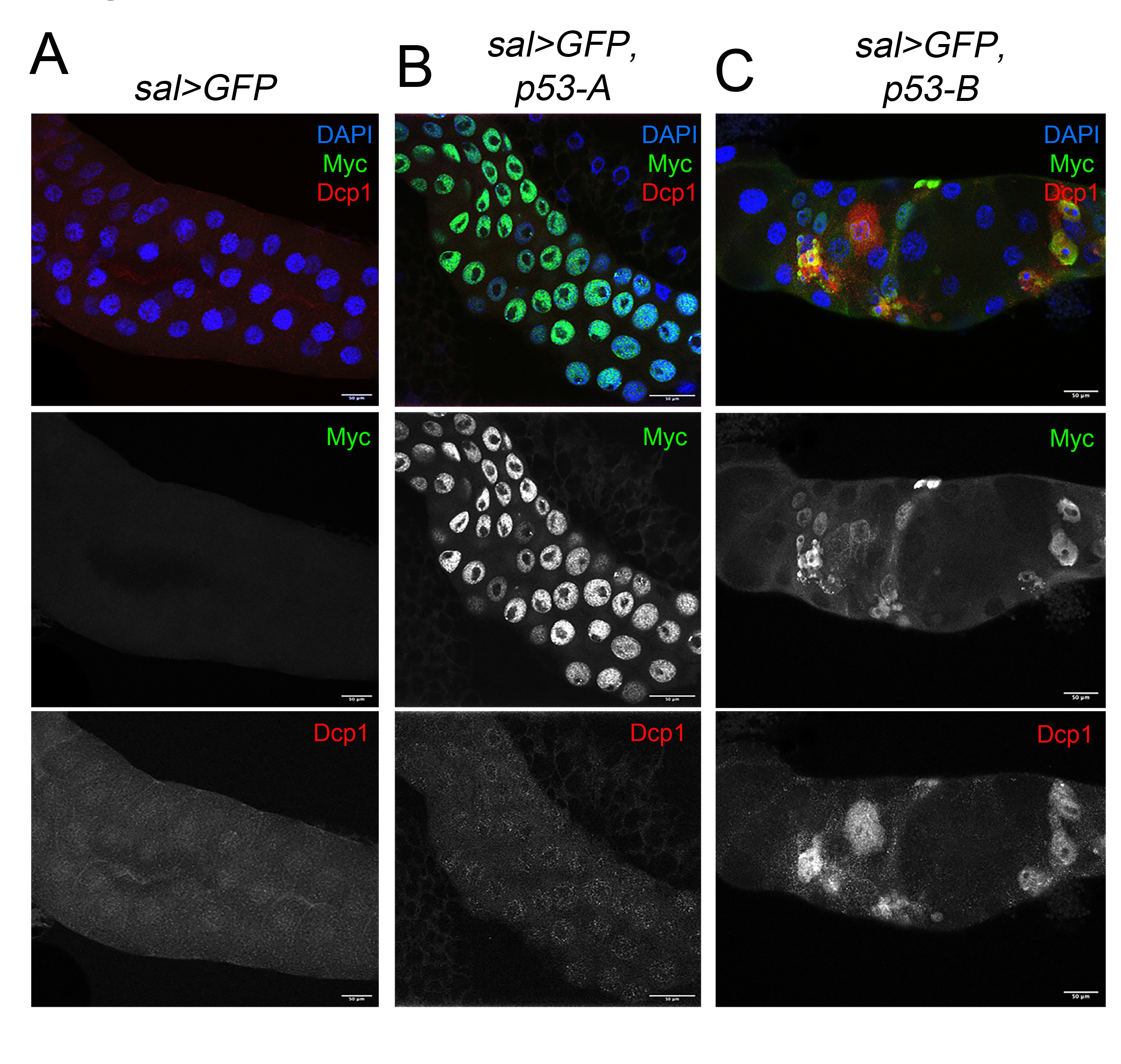

Supplement: Supplementary file 1 — Figure S1 [file 41419_2026_8571_MOESM1_ESM.tif]

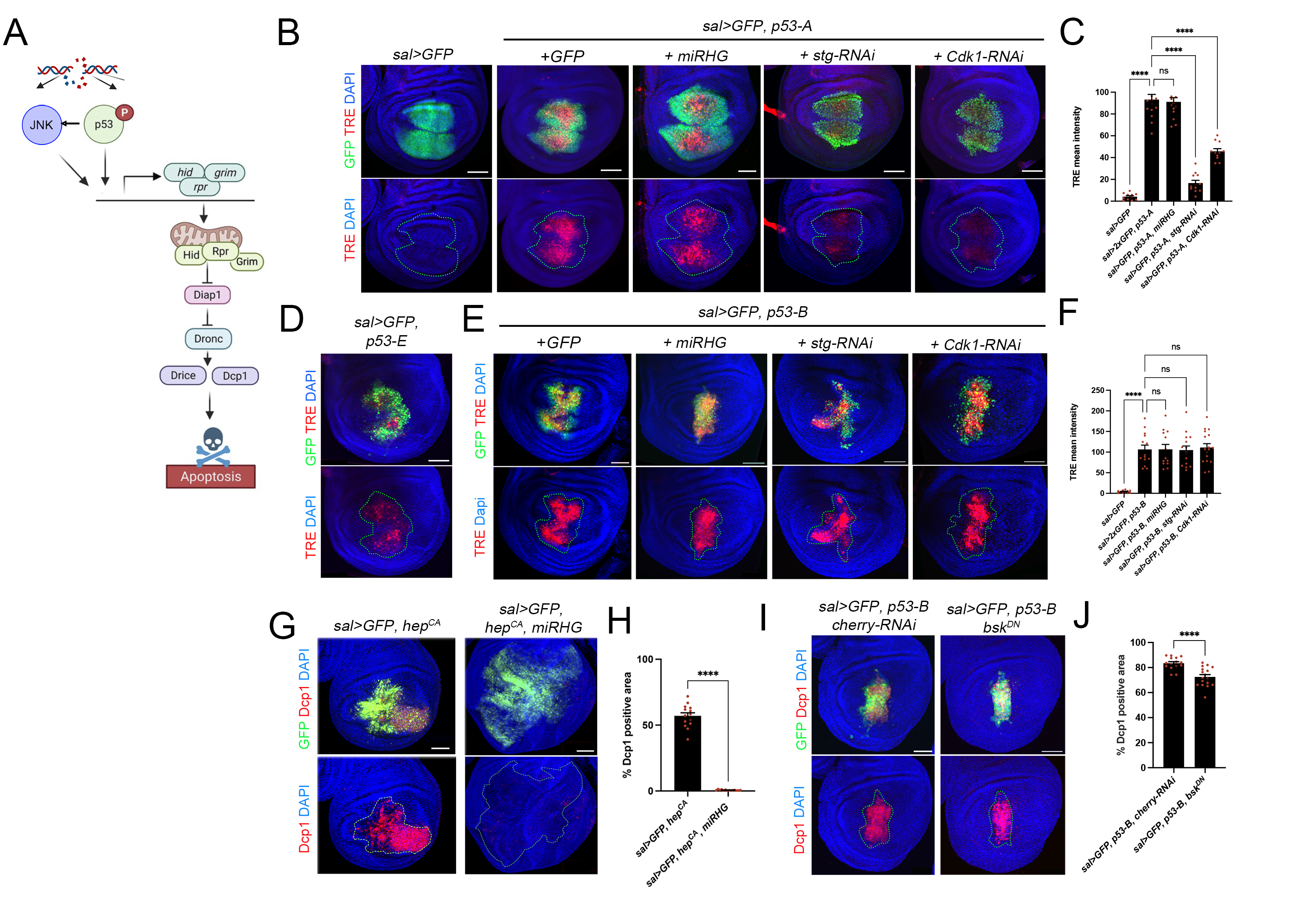

Supplement: Supplementary file 2 — Figure S2 [file 41419_2026_8571_MOESM2_ESM.tif]

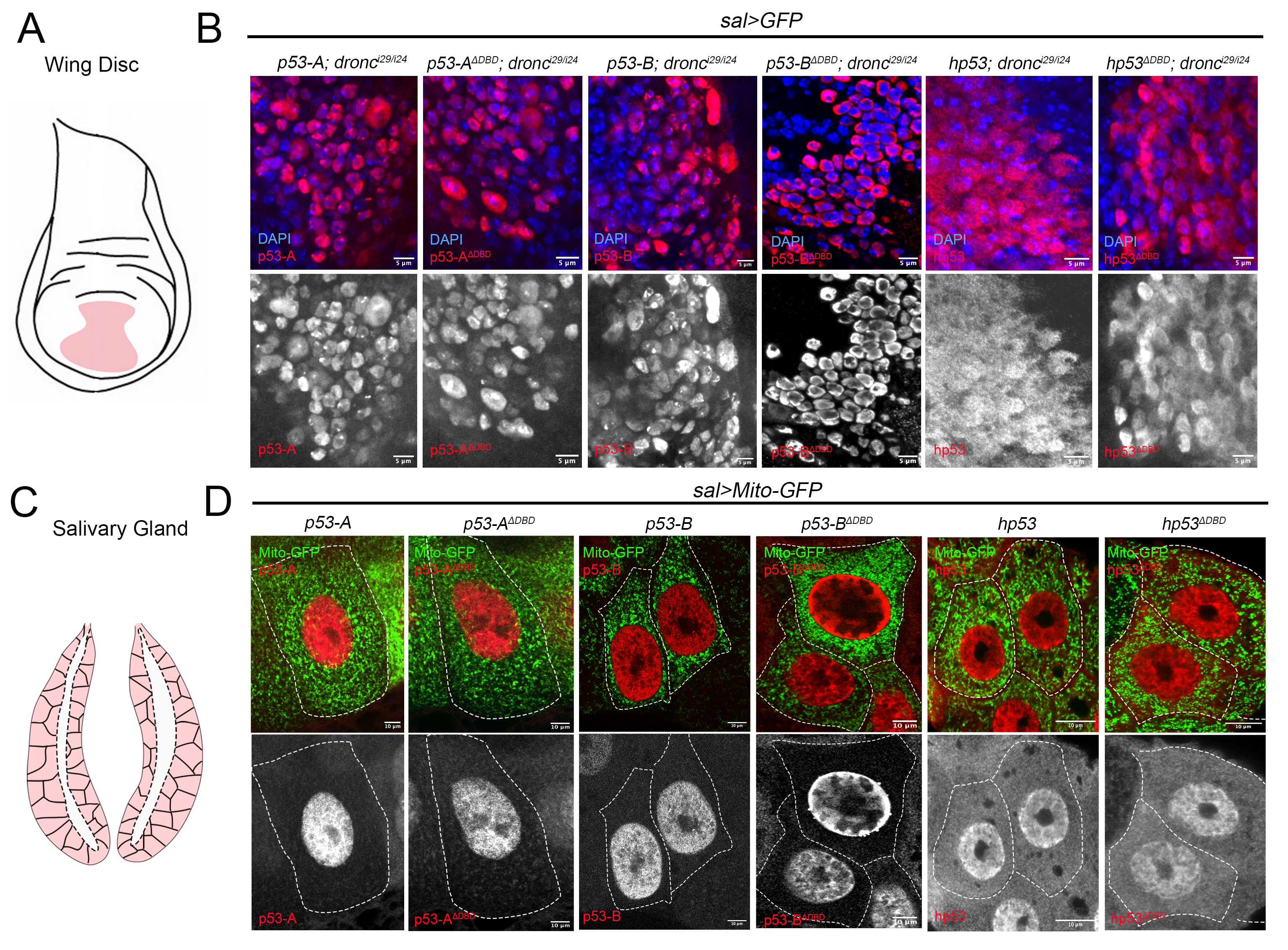

Supplement: Supplementary file 3 — Figure S3 [file 41419_2026_8571_MOESM3_ESM.tif]

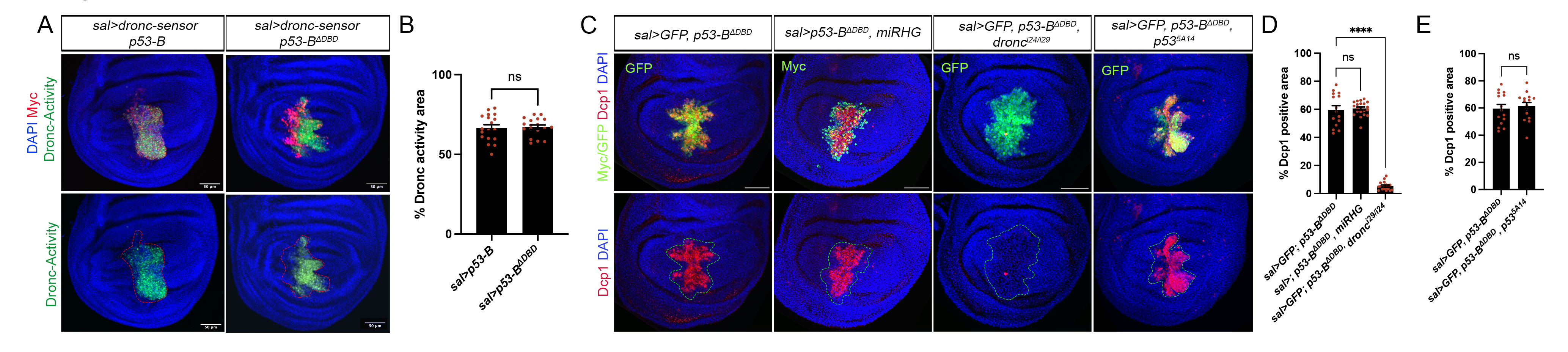

Supplement: Supplementary file 4 — Figure S4 [file 41419_2026_8571_MOESM4_ESM.tif]

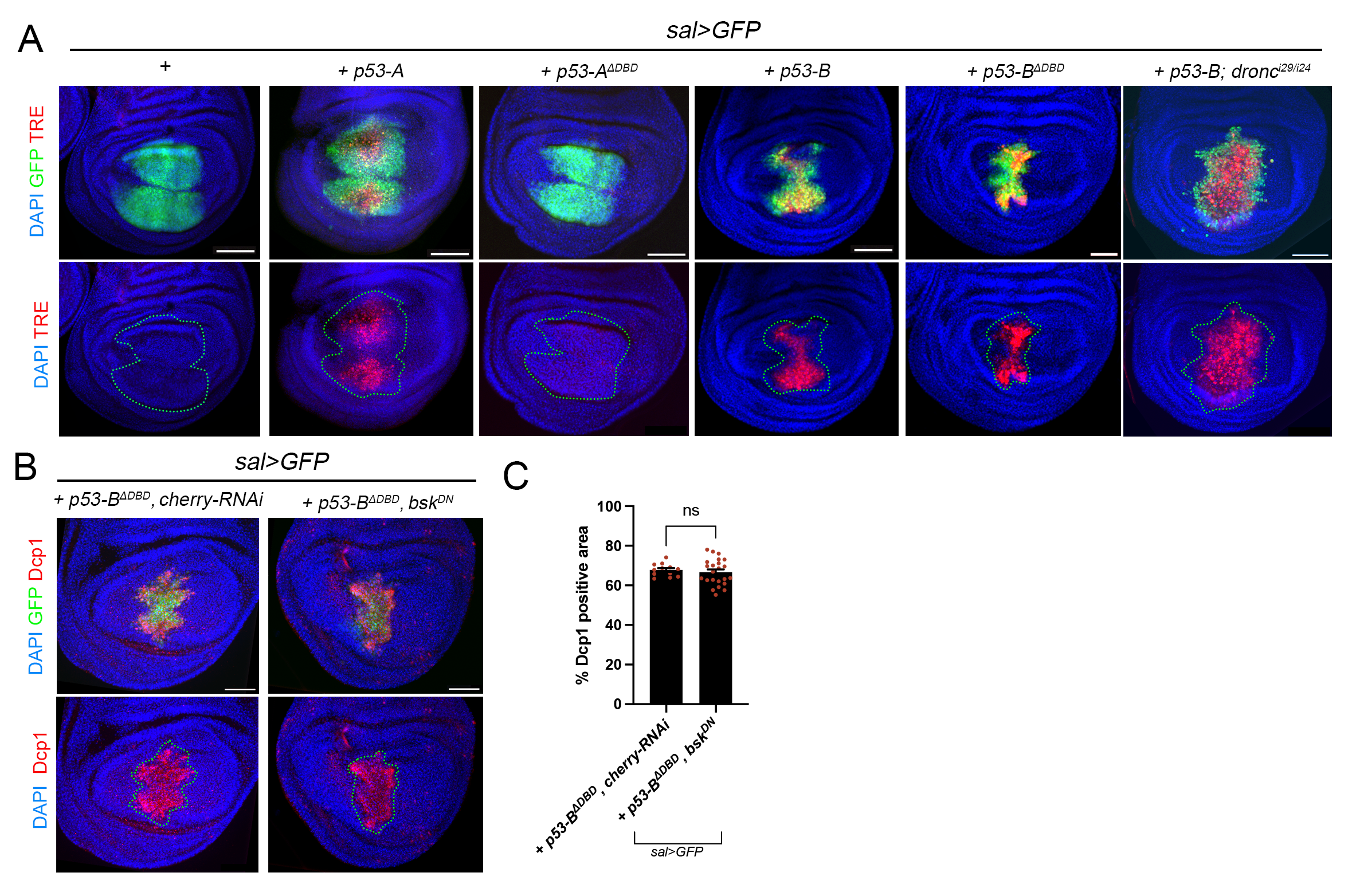

Supplement: Supplementary file 5 — Figure S5 [file 41419_2026_8571_MOESM5_ESM.tif]

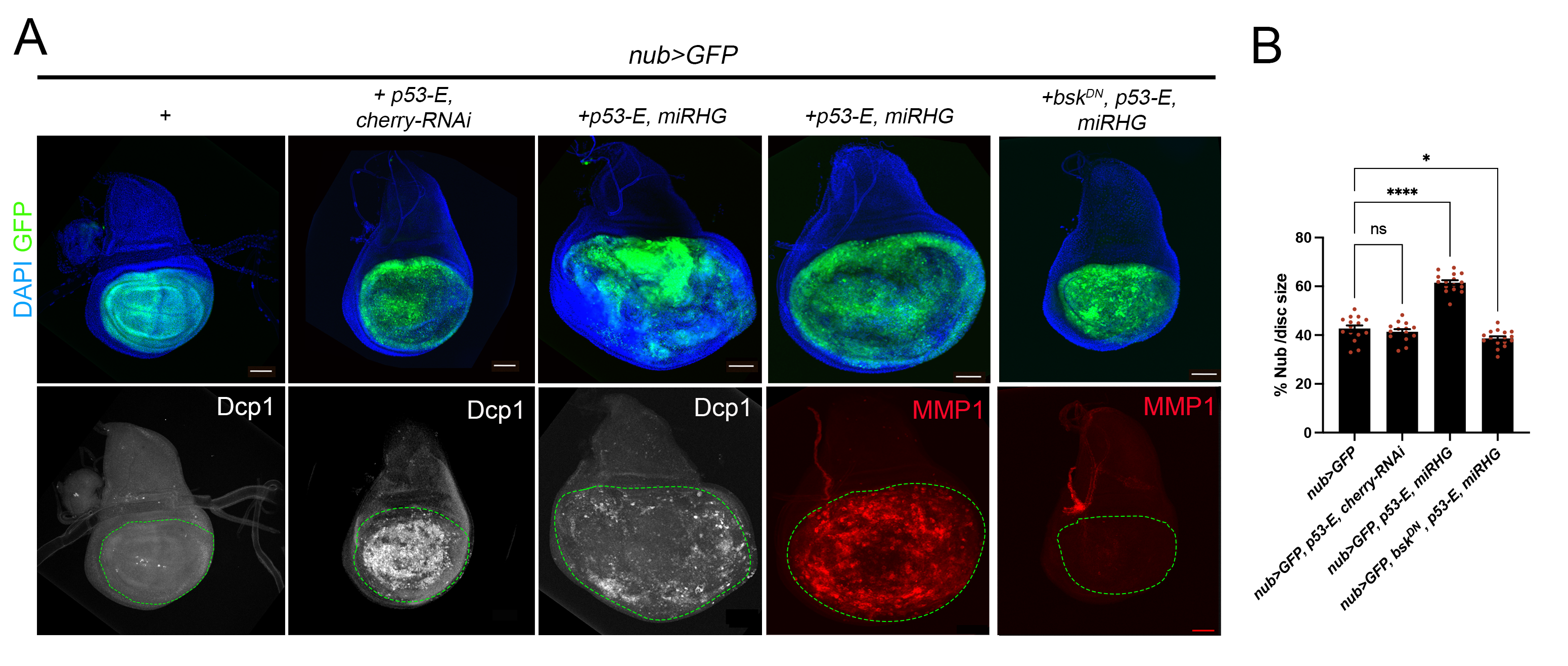

Supplement: Supplementary file 6 — Figure S6 [file 41419_2026_8571_MOESM6_ESM.tif]

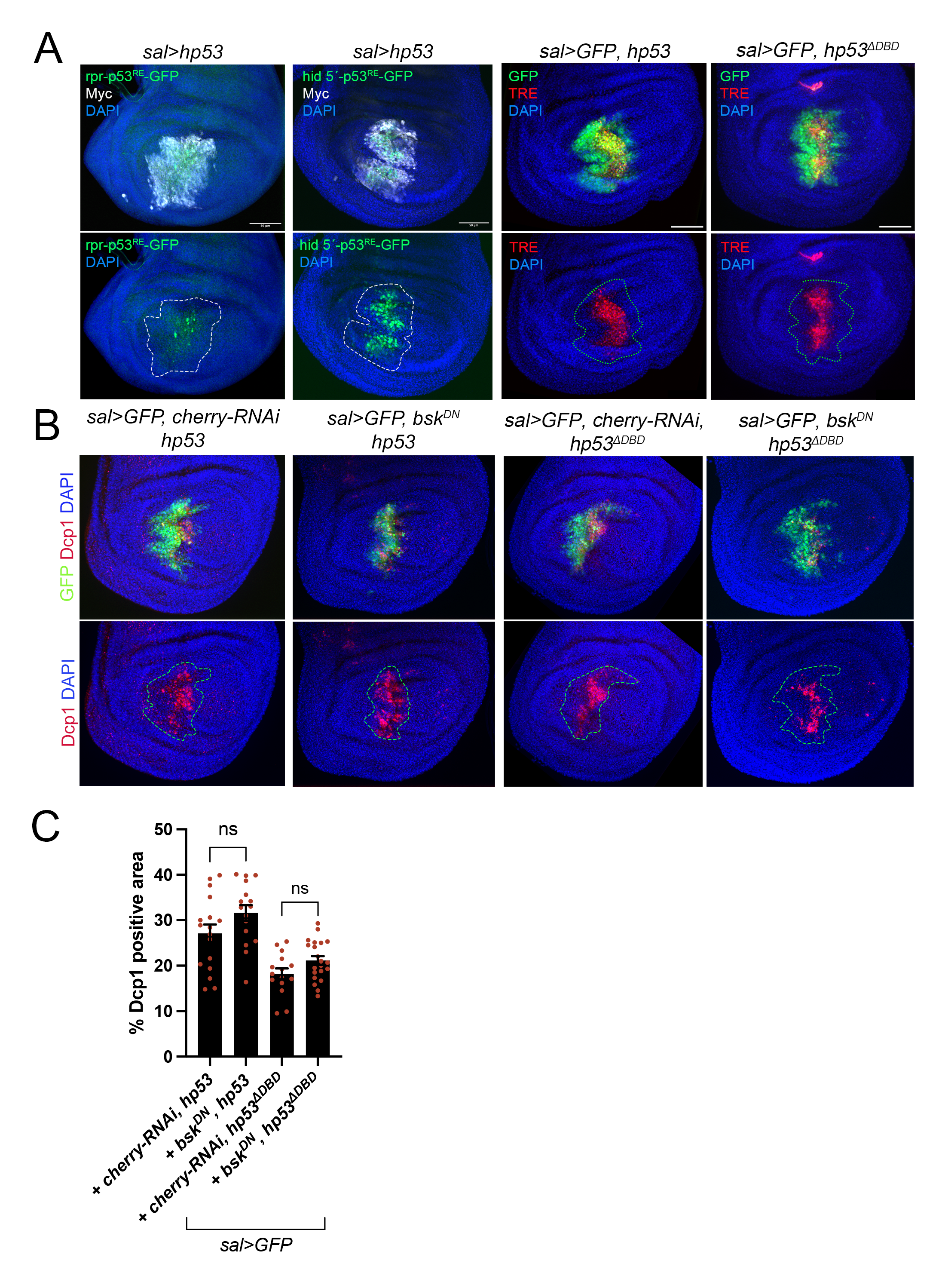

Supplement: Supplementary file 7 — Figure S7 [file 41419_2026_8571_MOESM7_ESM.tif]

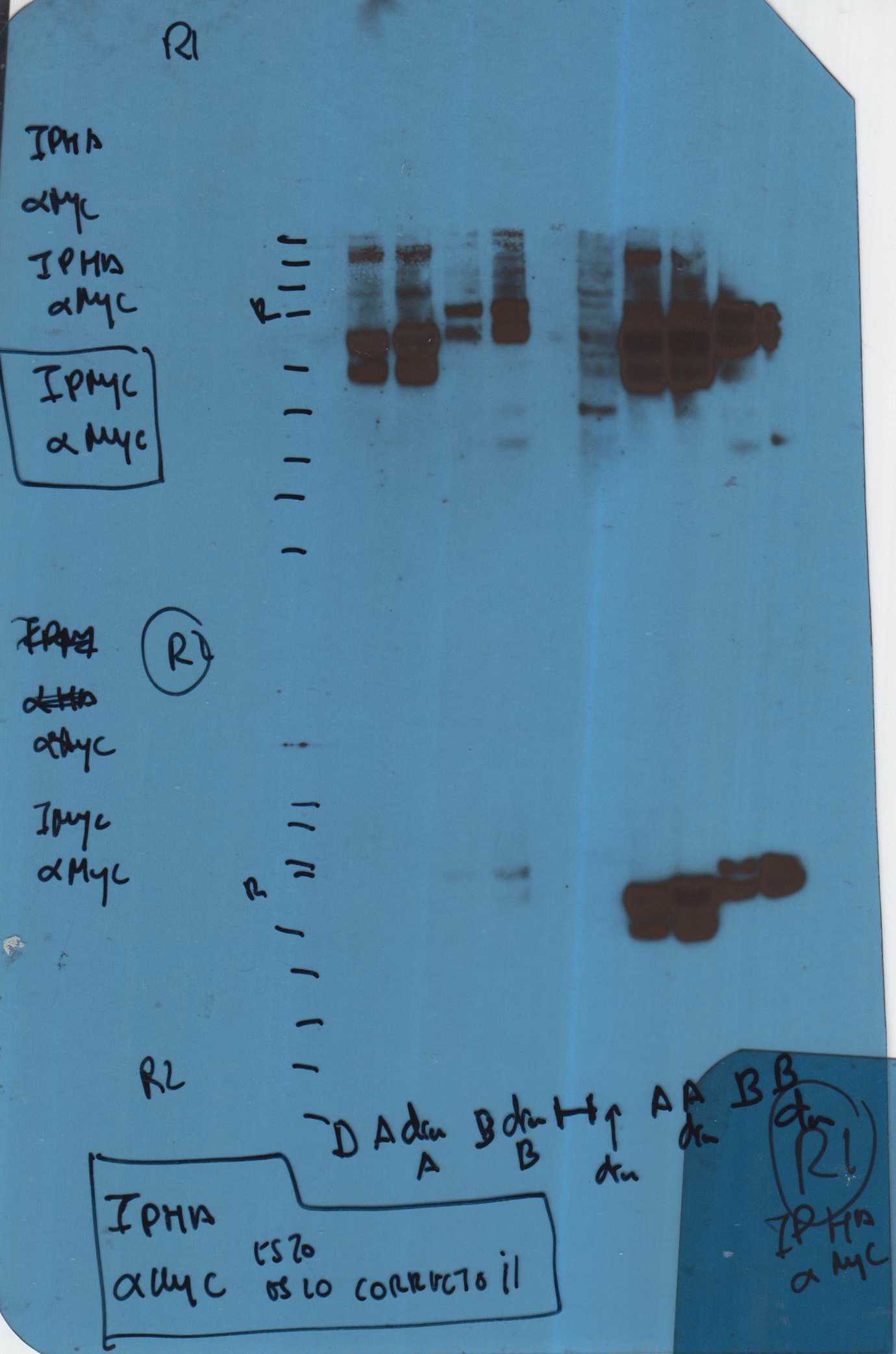

Supplement: Supplementary file 10 — Uncropped original western blot used in Figure 3H [file 41419_2026_8571_MOESM10_ESM.jpg]
