# Supplementary material for: Transcription-dependent and -independent functions of Drosophila p53 isoforms in the induction of apoptosis and senescence-associated tumorigenesis
Source: Cell Death Dis. 2026 Mar 25;17(1):367. doi: 10.1038/s41419-026-08571-x (PMC13039399; doi:10.1038/s41419-026-08571-x)
Supplement: Supplementary file 8 — Sup. Figure legends [file 41419_2026_8571_MOESM8_ESM.docx]

**Fig. S1:** **Expression of *p53-A* and *p53-B* in salivary glands.**

A-C) Representative salivary glands expressing the indicated transgenes and stained for Myc (green), DAPI (blue) and Dcp1 (red) under the *sal>* driver. Separate channels are shown in black and white. Note that the *sal-Gal4* driver is strongly expressed in the salivary glands. The scale bar is 50 μm.

**Fig. S2: Relationship between p53 isoforms and JNK pathway activation.**

A) Simplified representation of the apoptotic pathway triggered by p53 and its relationship with JNK. Created with [BioRender.com](http://BioRender.com).

B) Wing imaginal discs expressing the indicated transgenes under the *sal>* driver and stained for GFP (green), DAPI (blue) and TRE-RFP (red). The scale bar is 50 μm.

C) Quantification of TRE-RFP mean intensity (arbitrary units) in the *sal* domain of wing imaginal discs from the genotypes presented in B. The data are derived from three independent biological replicates, analyzing more than 10 discs per genotype. Error bars indicate SEM. ****P value < 0.0001 and not significant (ns) P value>0.05 by one-way ANOVA.

D and E) Wing imaginal discs expressing the indicated transgenes under the *sal>* driver and stained for GFP (green), DAPI (blue) and TRE-RFP (red). The scale bar is 50 μm.

F) Quantification of the mean intensity of TRE-RFP (arbitrary units) in the *sal* domain of wing imaginal discs from the genotypes presented in E. The data are derived from three independent biological replicates, analyzing more than 10 discs per genotype. Error bars indicate SEM. ****P value < 0.0001 and not significant (ns) P value>0.05 by one-way ANOVA.

G) Wing imaginal discs expressing UAS-*hep^CA^* alone or in combination with the UAS-*miRHG* under the *sal>GFP* driver and stained for GFP (green), DAPI (blue) and Dcp1(red). Scale bar is 50 μm.

H) Quantification of Dcp1 staining in the *sal* domain of wing imaginal discs from the genotypes presented in G. The data are derived from three independent biological replicates, analyzing more than 12 discs per genotype. Error bars indicate SEM. ****P value < 0.0001 by unpaired t test.

I) Wing imaginal discs expressing UAS-*p53-B* in combination with the indicated transgenes under the *sal>GFP* driver and stained for GFP (green), DAPI (blue) and Dcp1(red). The scale bar is 50 μm.

J) Quantification of Dcp1 staining in the *sal* domain of wing imaginal discs from the genotypes presented in I. The data are derived from three independent biological replicates, analyzing more than 12 discs per genotype. Error bars indicate SEM. ****P value < 0.0001 by unpaired t-test.

**Fig. S3: Cellular localization of p53-A, p53-B and hp53 versions with and without the DBD.**

A and C) Cartoons of a wing imaginal disc (A) and of a salivary gland (C) with the domain of the sal-Gal4 driver in pink.

B and D) Close-up views of wing imaginal discs (B) and of a salivary gland (D) expressing the indicated transgenes and stained for Myc (red) and DAPI (blue) in B and for Myc (red) and Mito-GFP (green) in D. Scale bar is 5 μm in B and 10 μm in D.

**Fig. S4: Apoptotic induction by p53-B^ΔDBD^.**

A) Wing imaginal discs expressing the Dronc activity sensor under the *sal>* driver and the corresponding transgenes. The imaginal discs were stained for GFP (green), Myc (red) and DAPI (blue). A dotted red line marks the sal domain delimited by Myc staining. The scale bar is 50 μm.

B) Quantification of GFP (Dronc activity) in the *sal* domain of wing imaginal discs from the genotypes presented in A. The data are derived from three independent biological replicates, analyzing more than 15 discs per genotype. Error bars indicate SEM. Not significant (ns) P value>0.05 by unpaired t-test.

C) Wing imaginal discs expressing UAS-*p53-B^ΔDBD^* in combination with the indicated transgenes or mutant backgrounds under the *sal>GFP* driver and stained for GFP (green), DAPI (blue) and Dcp1(red). A dotted green line marks the *sal* domain. The scale bar is 50 μm.

D and E) Quantification of Dcp1 staining in the *sal* domain of wing imaginal discs from the genotypes presented in B. The data are derived from three independent biological replicates, analyzing more than 12 discs per genotype. Error bars indicate SEM. ****P value < 0.0001 and not significant (ns) P value>0.05 by one-way ANOVA in D and not significant (ns) P value>0.05 by unpaired t-test in E.

**Fig. S5: Activation of the JNK pathway by p53-A^ΔDBD^ and p53-B^ΔDBD^.**

A) Representative wing imaginal discs expressing the indicated transgenes and mutant backgrounds under the *sal>GFP* driver (n>10) and stained for GFP (green), DAPI (blue) and TRE-RFP (red). A dotted green line marks the *sal* domain. The scale bar is 50 μm.

B) Wing imaginal discs expressing the indicated transgenes under the *sal>GFP* driver and stained for GFP (green), DAPI (blue) and Dcp1 (red). A dotted green line marks the *sal* domain. The scale bar is 50 μm.

C) Quantification of Dcp1 staining in the *sal* domain of wing imaginal discs from the genotypes presented in B. The data are derived from three independent biological replicates, analyzing more than 10 discs per genotype. Error bars indicate SEM. Not significant (ns) P value>0.05 by unpaired t-test.

**Fig. S6: p53-E induces tumor overgrowths in apoptosis deficient cells.**

A) Representative wing imaginal discs expressing *p53-E* using the *nub>* driver (n>10). When indicated the over-expression of *p53-E* was combined with the UAS-*miRHG* or performed in a *dronc* mutant background. Imaginal discs were stained for GFP (green), DAPI (blue) and Dcp1 (white) or MMP1 (red). A dotted green line marks the *nub* domain. The scale bar is 50 μm.

B) Quantification of the tumor overgrowths from the genotypes indicated in A calculated as a percentage of the *nub* domain. The data are derived from three independent biological replicates, analyzing more than 10 discs per genotype. Error bars indicate SEM. ****P value < 0.0001, *P value < 0.05 and not significant (ns) P value>0.05 by one-way ANOVA.

**Fig. S7: Activation of *hid* 5′-p53^RE^-GFP, *rpr-*p53^RE^-GFP and TRE-RFP reporters by *hp53.***

A) Representative wing imaginal discs expressing the indicated transgenes with the *sal>* driver and stained for the activity of the *hid* 5′-p53^RE^-GFP, *rpr* 5′-p53^RE^-GFP and TRE-RFP (n>10). Myc (white), GFP (green) and DAPI (blue) channels are shown when indicated. A dotted line marks the *sal* domain. The scale bar is 50 μm.

B) Wing imaginal discs expressing the indicated transgenes with the *sal>GFP* driver stained for GFP (green), DAPI (blue) and Dcp1 (red). A dotted green line marks the *sal* domain. The scale bar is 50 μm.

C) Quantification of Dcp1 staining in the *sal* domain of wing imaginal discs from the genotypes presented in B. The data are derived from three independent biological replicates, analyzing more than 10 discs per genotype. Error bars indicate SEM. not significant (ns) P value>0.05 by one-way ANOVA.

**Table S1:** Complete list of the genotypes used for each figure.
