# Supplementary material for: Transcription-dependent and -independent functions of Drosophila p53 isoforms in the induction of apoptosis and senescence-associated tumorigenesis
Source: Cell Death Dis. 2026 Mar 25;17(1):367. doi: 10.1038/s41419-026-08571-x (PMC13039399; doi:10.1038/s41419-026-08571-x)
Supplement: Supplementary file 9 — Suplementary Table 1 [file 41419_2026_8571_MOESM9_ESM.docx]

| Figure 1: | Genotype |
| --- | --- |
| 1C | *+/*(*+*)*; sal>UAS-GFP/+; +/+* |
|  | *+/*(*+*)*; sal>UAS-GFP/+; cherry-RNAi/UAS-6xMyc:p53-A* |
|  | *+/*(*+*)*; sal>UAS-GFP/UAS-dap; +/UAS-6xMyc:p53-A* |
|  | *+/*(*+*)*; sal>UAS-GFP/UAS-stg-RNAi; +/UAS-6xMyc:p53-A* |
|  | *+/*(*+*)*; sal>UAS-GFP/UAS-Cdk1-RNAi; +/UAS-6xMyc:p53-A* |
|  | *+/*(*+*)*; sal>UAS-GFP/+; UAS-fzr/UAS-6xMyc:p53-A* |
| 1E | *+/*(*+*)*; sal>UAS-GFP/+; +/+* |
|  | *+/*(*+*)*; sal>UAS-GFP/+; cherry-RNAi/UAS-6xMyc:p53-B* |
|  | *+/*(*+*)*; sal>UAS-GFP/UAS-dap; +/UAS-6xMyc:p53-B* |
|  | *+/*(*+*)*; sal>UAS-GFP/UAS-stg-RNAi; +/UAS-6xMyc:p53-B* |
|  | *+/*(*+*)*; sal>UAS-GFP/UAS-Cdk1-RNAi; +/UAS-6xMyc:p53-B* |
|  | *+/*(*+*)*; sal>UAS-GFP/+; UAS-fzr/UAS-6xMyc:p53-B* |
| 1G | *+/*(*+*)*; sal>UAS-GFP/+; +/+* |
|  | *+/*(*+*)*; sal>UAS-GFP/+; cherry-RNAi/UAS-6xMyc:p53-E* |
|  | *+/*(*+*)*; sal>UAS-GFP/UAS-dap; +/UAS-6xMyc:p53-E* |
|  | *+/*(*+*)*; sal>UAS-GFP/UAS-stg-RNAi; +/UAS-6xMyc:p53-E* |
|  | *+/*(*+*)*; sal>UAS-GFP/UAS-Cdk1-RNAi; +/UAS-6xMyc:p53-E* |
|  | *+/*(*+*)*; sal>UAS-GFP/+; UAS-fzr/UAS-6xMyc:p53-E* |
| Figure 2: | Genotype |
| 2B | *+/*(*+*)*; sal>UAS-GFP/+; UAS-6xMyc:p53-A/+* |
|  | *+/*(*+*)*; sal>/UAS-miRHG; UAS-6xMyc:p53-A/+* |
|  | *+/*(*+*)*; sal>UAS-GFP/+; UAS-6xMyc:p53-E/+* |
|  | *+/*(*+*)*; sal>/UAS-miRHG; UAS-6xMyc:p53-E/+* |
|  | *+/*(*+*)*; sal>UAS-GFP/+; UAS-6xMyc:p53-B/+* |
|  | *+/*(*+*)*; sal>/UAS-miRHG; UAS-6xMyc:p53-B/+* |
| 2D | *yw hs-flp tub-gal4, UAS-GFP; UAS-p53-A:6xMYC/+; tub-Gal80 FRT2A/ FRT2A* |
|  | *yw hs-flp tub-gal4, UAS-GFP; UAS-p53-A:6xMYC/+; tub-Gal80 FRT2A/DfH99 FRT2A* |
|  | *yw hs-flp tub-gal4, UAS-GFP; UAS-p53-B:6xMYC/+; tub-Gal80 FRT2A/ FRT2A* |
|  | *yw hs-flp tub-gal4, UAS-GFP; UAS-p53-B:6xMYC/+; tub-Gal80 FRT2A/DfH99 FRT2A* |
| Figure 3: | Genotype |
| 3A | *+/*(*+*)*; sal>UAS-GFP/+; UAS-6xMyc:p53-A/UAS-GFP* |
|  | *+/*(*+*)*; sal>UAS-GFP/+; UAS-6xMyc:p53-A/UAS-Diap1* |
|  | *+/*(*+*)*; sal>UAS-GFP/+; UAS-6xMyc:p53-B/UAS-GFP* |
|  | *+/*(*+*)*; sal>UAS-GFP/+; UAS-6xMyc:p53-B/UAS-Diap1* |
| 3C | *+/*(*+*)*; sal>UAS-GFP/+; UAS-6xMyc:p53-A/+* |
|  | *+/*(*+*)*; sal>UAS-GFP/+; UAS-6xMyc:p53-A, dronc^i29^/dronc^i24^* |
|  | *+/*(*+*)*; sal>UAS-GFP/+; UAS-6xMyc:p53-B/+* |
|  | *+/*(*+*)*; sal>UAS-GFP/+; UAS-6xMyc:p53-B, dronc^i29^/dronc^i24^* |
| 3F | *+/*(*+*)*; sal>/UAS-dronc-GFP-TETDG-Myc; +/+* |
|  | *+/*(*+*)*; sal>/UAS-dronc-GFP-TETDG-Myc; UAS-6xMyc:p53-A/+* |
|  | *+/*(*+*)*; sal>UAS-miRHG /* *UAS-dronc-GFP-TETDG-Myc; UAS-6xMyc:p53-A/+* |
|  | *+/*(*+*)*; sal>UAS-dronc-GFP-TETDG-Myc; UAS-6xMyc:p53-B/+* |
|  | *+/*(*+*)*; sal>UAS-miRHG /* *UAS-dronc-GFP-TETDG-Myc; UAS-6xMyc:p53-B/+* |
| 3H | *+/*(*+*)*; +/+; dronc-HA/+* |
|  | *+/*(*+*)*; sal>UAS-GFP/+; UAS-6xMyc:p53-A/+* |
|  | *+/*(*+*)*; sal>UAS-GFP/+; UAS-6xMyc:p53-A/dronc-HA* |
|  | *+/*(*+*)*; sal>UAS-GFP/+; UAS-6xMyc:p53-B/+* |
|  | *+/*(*+*)*; sal>UAS-GFP/+; UAS-6xMyc:p53-B/dronc-HA* |
| 3K | *+/*(*+*)*; sal>/+; UAS-VN-p53-B /+* |
|  | *+/*(*+*)*; sal>/+; UAS-dronc-VC /+* |
|  | *+/*(*+*)*; sal>/+; UAS-VN-p53-B/UAS-dronc-VC* |
|  | *+/*(*+*)*; sal>/+; UAS-VN-p53-B^ΔTAD^/UAS-dronc-VC* |
| Figure 4: | Genotype |
| 4A | *+/*(*+*)*; sal>, hid-5´-p53^RE^-GFP/+; +/+* |
|  | *+/*(*+*)*; sal>, hid-5´-p53^RE^-GFP/+; UAS-p53-A:6xMyc/+* |
|  | *+/*(*+*)*; sal>, hid-5´-p53^RE^-GFP/+; UAS-p53-A^ΔDBD^:6xMyc/+* |
| 4B | *+/*(*+*)*; sal>, hid-5´-p53^RE^-GFP/+; UAS-p53-B:6xMyc/+* |
|  | *+/*(*+*)*; sal>, hid-5´-p53^RE^-GFP/+; UAS-p53-B^ΔDBD^:6xMyc/+* |
| 4C | *+/*(*+*)*; sal>, rpr-p53^RE^-GFP/+; +/+* |
|  | *+/*(*+*)*; sal>, rpr-p53^RE^-GFP/+; UAS-p53-A:6xMyc/+* |
|  | *+/*(*+*)*; sal>, rpr-p53^RE^-GFP/+; UAS-p53-A^ΔDBD^:6xMyc/+* |
| 4D | *+/*(*+*)*; sal>, rpr-p53^RE^-GFP/+; UAS-p53-B:6xMyc/+* |
|  | *+/*(*+*)*; sal>, rpr-p53^RE^-GFP/+; UAS-p53-B^ΔDBD^:6xMyc/+* |
| 4E | *+/*(*+*)*; sal>UAS-GFP/+; +/+* |
|  | *+/*(*+*)*; sal>/+; UAS-p53-A:6xMyc/+* |
|  | *+/*(*+*)*; sal>/+; UAS-p53-A^ΔDBD^:6xMyc/+* |
| 4F | *+/*(*+*)*; sal>/+; UAS-p53-B:6xMyc/+* |
|  | *+/*(*+*)*; sal>/+; UAS-p53-B^ΔDBD^:6xMyc/+* |
| Figure 5 | Genotype |
| 5A | *+/*(*+*)*; nub>UAS-GFP/+; +/+* |
|  | *+/*(*+*)*; nub>UAS-GFP/+; UAS-cherry-RNAi/ UAS-p53-A:6xMyc* |
|  | *+/*(*+*)*; nub>UAS-GFP/UAS-miRHG; UAS-p53-A:6xMyc/+* |
|  | *+/*(*+*)*; nub>UAS-GFP/UAS-p53-A:6xMyc; dronc^i29^/dronc^i24^* |
|  | *+/*(*+*)*; nub>UAS-GFP/UAS-p53-A^ΔDBD^:6xMyc; dronc^i29^/dronc^i24^* |
| 5C | *+/*(*+*)*; nub>UAS-GFP/+; +/+* |
|  | *+/*(*+*)*; nub>UAS-GFP/UAS-p53-A:6xMyc; dronc^i29^/dronc^i24^* |
|  | *UAS-bsk^DN^/+; nub>UAS-GFP/UAS-p53-A:6xMyc; dronc^i29^/dronc^i24^* |
|  | *+/*(*+*)*; nub>UAS-GFP/UAS-p53-A^ΔDBD^:6xMyc; dronc^i29^/dronc^i24^* |
| 5E | *+/*(*+*)*; nub>UAS-GFP/+; +/+* |
|  | *+/*(*+*)*; nub>UAS-GFP/+; UAS-cherry-RNAi/ UAS-p53-B:6xMyc* |
|  | *+/*(*+*)*; nub>UAS-GFP/UAS-miRHG; UAS-p53-B:6xMyc/+* |
|  | *+/*(*+*)*; nub>UAS-GFP/UAS-p53-B:6xMyc; dronc^i29^/dronc^i24^* |
|  | *+/*(*+*)*; nub>UAS-GFP/UAS-p53-B^ΔDBD^:6xMyc; dronc^i29^/dronc^i24^* |
| 5G | *+/*(*+*)*; nub>UAS-GFP/+; +/+* |
|  | *+/*(*+*)*; nub>UAS-GFP/UAS-p53-B:6xMyc; dronc^i29^/dronc^i24^* |
|  | *UAS-bsk^DN^/+; nub>UAS-GFP/UAS-p53-B:6xMyc; dronc^i29^/dronc^i24^* |
|  | *+/*(*+*)*; nub>UAS-GFP/UAS-p53-B^ΔDBD^:6xMyc; dronc^i29^/dronc^i24^* |
| Figure 6 | Genotype |
| 6A | *+/*(*+*)*; sal>UAS-GFP/+; +/+* |
|  | *+/*(*+*)*; sal>UAS-GFP/+; UAS-hp53:6xMyc/+* |
|  | *+/*(*+*)*; sal>UAS-miRHG/+; UAS-hp53:6xMyc/+* |
|  | *+/*(*+*)*; sal>UAS-GFP/ UAS-hp53:6xMyc; dronc^i29^/dronc^i24^* |
| 6C | *+/*(*+*)*; sal>UAS-GFP/UAS-dap; UAS-hp53:6xMyc/+* |
|  | *+/*(*+*)*; sal>UAS-GFP/+; UAS-hp53:6xMyc/UAS-stg-RNAi* |
|  | *+/*(*+*)*; sal>UAS-GFP/+; UAS-hp53:6xMyc/UAS-Cdk1-RNAi* |
|  | *+/*(*+*)*; sal>UAS-GFP/+; UAS-hp53:6xMyc/UAS-fzr* |
| 6E | *+/*(*+*)*; sal>/UAS-dronc-GFP-TETDG-Myc; +/+* |
|  | *+/*(*+*)*; sal>/UAS-dronc-GFP-TETDG-Myc; UAS-6xMyc:hp53/+* |
|  | *+/*(*+*)*; sal>UAS-miRHG /* *UAS-dronc-GFP-TETDG-Myc; UAS-6xMyc:hp53^ΔDBD^/+* |
| 6F | *+/*(*+*)*; sal>UAS-GFP/UAS-hp53:6xMyc; +/+* |
|  | *+/*(*+*)*; sal>UAS-GFP/UAS-hp53^ΔDBD^:6xMyc; +/+* |
|  | *+/*(*+*)*; sal>UAS-GFP/UAS-hp53^ΔDBD^:6xMyc; dronc^i29^/dronc^i24^* |
|  | *+/*(*+*)*; sal>UAS-GFP/UAS-hp53^ΔDBD^:6xMyc; p53^5A14^/p53^5A14^* |
| Figure 7 | Genotype |
| 7A | *+/*(*+*)*; nub>UAS-GFP/+; +/+* |
|  | *+/*(*+*)*; nub>UAS-GFP/+; UAS-cherry-RNAi/ UAS-hp53:6xMyc* |
|  | *+/*(*+*)*; nub>UAS-GFP/UAS-miRHG; UAS-hp53:6xMyc /+* |
|  | *+/*(*+*)*; nub>UAS-GFP/ UAS-hp53:6xMyc; dronc^i29^/dronc^i24^* |
| 7C and 7D | *+/*(*+*)*; nub>UAS-GFP/+; +/+* |
|  | *+/*(*+*)*; nub>UAS-GFP/ UAS-hp53:6xMyc; dronc^i29^/dronc^i24^* |
| Figure S1 | Genotype |
|  | *+/*(*+*)*; sal>UAS-GFP/+; +/+* |
|  | *+/*(*+*)*; sal>UAS-GFP/+; UAS-6xMyc:p53-A /+* |
|  | *+/*(*+*)*; sal>UAS-GFP/+; UAS-6xMyc:p53-B /+* |
| Figure S2 | Genotype |
| S2B | *+/*(*+*)*; sal>UAS-GFP, TRE-RFP/+; +/+* |
|  | *+/*(*+*)*; sal>UAS-GFP, TRE-RFP/UAS-GFP; UAS-6xMyc:p53-A/+* |
|  | *+/*(*+*)*; sal>UAS-GFP, TRE-RFP/UAS-miRHG; UAS-6xMyc:p53-A/+* |
|  | *+/*(*+*)*; sal>UAS-GFP, TRE-RFP/+; UAS-6xMyc:p53-A/UAS-stg-RNAi* |
|  | *+/*(*+*)*; sal>UAS-GFP, TRE-RFP/+; UAS-6xMyc:p53-A/UAS-Cdk1-RNAi* |
| S2D | *+/*(*+*)*; sal>UAS-GFP, TRE-RFP/+; UAS-6xMyc:p53-E/+* |
| S2E | *+/*(*+*)*; sal>UAS-GFP, TRE-RFP/UAS-GFP; UAS-6xMyc:p53-B/+* |
|  | *+/*(*+*)*; sal>UAS-GFP, TRE-RFP/UAS-miRHG; UAS-6xMyc:p53-B/+* |
|  | *+/*(*+*)*; sal>UAS-GFP, TRE-RFP/+; UAS-6xMyc:p53-B/UAS-stg-RNAi* |
|  | *+/*(*+*)*; sal>UAS-GFP, TRE-RFP/+; UAS-6xMyc:p53-B/UAS-Cdk1-RNAi* |
| S2G | *+/*(*+*)*; sal>UAS-GFP/+; UAS-hep^CA^/+* |
|  | *+/*(*+*)*; sal>UAS-GFP/UAS-miRHG; UAS-hep^CA^/+* |
| S2I | *+/*(*+*)*; sal>UAS-GFP/+; UAS-6xMyc:p53-B/UAS-cherry-RNAi* |
|  | *UAS-bsk^DN^/+; sal>UAS-GFP/+; UAS-6xMyc:p53-B/+* |
| Figure S3 | Genotype |
| S3B | *+/*(*+*)*; sal>UAS-GFP/UAS-p53-A:6xMyc; dronc^i29^/dronc^i24^* |
|  | *+/*(*+*)*; sal>UAS-GFP/UAS-p53-A^ΔDBD^:6xMyc; dronc^i29^/dronc^i24^* |
|  | *+/*(*+*)*; sal>UAS-GFP/UAS-p53-B:6xMyc; dronc^i29^/dronc^i24^* |
|  | *+/*(*+*)*; sal>UAS-GFP/UAS-p53-B^ΔDBD^:6xMyc; dronc^i29^/dronc^i24^* |
|  | *+/*(*+*)*; sal>UAS-GFP/UAS-hp53:6xMyc; dronc^i29^/dronc^i24^* |
|  | *+/*(*+*)*; sal>UAS-GFP/UAS-hp53^ΔDBD^:6xMyc; dronc^i29^/dronc^i24^* |
| S3D | *+/*(*+*)*; sal>/UAS-p53-A:6xMyc; UAS-mito:GFP/+* |
|  | *+/*(*+*)*; sal>/UAS-p53-A^ΔDBD^:6xMyc; UAS-mito:GFP/+* |
|  | *+/*(*+*)*; sal>/UAS-p53-B:6xMyc; UAS-mito:GFP/+* |
|  | *+/*(*+*)*; sal>/UAS-p53-B^ΔDBD^:6xMyc; UAS-mito:GFP/+* |
|  | *+/*(*+*)*; sal>/UAS-hp53:6xMyc; UAS-mito:GFP/+* |
|  | *+/*(*+*)*; sal>/UAS-hp53^ΔDBD^:6xMyc; UAS-mito:GFP/+* |
| Figure S4 | Genotype |
| S4A | *+/*(*+*)*; sal>UAS-dronc-GFP-TETDG-Myc; UAS-6xMyc:p53-B/+* |
|  | *+/*(*+*)*; sal>UAS-dronc-GFP-TETDG-Myc; UAS-6xMyc:p53-B^ΔDBD^/+* |
| S4C | *+/*(*+*)*; sal>UAS-GFP/UAS-p53-B^ΔDBD^:6xMyc; +/+* |
|  | *+/*(*+*)*; sal>UAS-miRHG/UAS-p53-B^ΔDBD^:6xMyc; +/+* |
|  | *+/*(*+*)*; sal>UAS-GFP/UAS-p53-B^ΔDBD^:6xMyc; dronc^i29^/dronc^i24^* |
|  | *+/*(*+*)*; sal>UAS-GFP/UAS-p53-B^ΔDBD^:6xMyc; p53^5A14^/p53^5A14^* |
| Figure S5 | Genotype |
| S5A | *+/*(*+*)*; sal>UAS-GFP, TRE-RFP/+; +/+* |
|  | *+/*(*+*)*; sal>UAS-GFP, TRE-RFP/+; UAS-p53-A:6xMyc/+* |
|  | *+/*(*+*)*; sal>UAS-GFP, TRE-RFP/+; UAS-p53-A^ΔDBD^:6xMyc/+* |
|  | *+/*(*+*)*; sal>UAS-GFP, TRE-RFP/+; UAS-p53-B:6xMyc/+* |
|  | *+/*(*+*)*; sal>UAS-GFP, TRE-RFP/+; UAS-p53-B^ΔDBD^:6xMyc/+* |
|  | *+/*(*+*)*; sal>UAS-GFP, TRE-RFP/UAS-p53-B:6xMyc; dronc^i29^/dronc^i24^* |
| S5B | *+/*(*+*)*; sal>UAS-GFP/+; UAS-p53-B^ΔDBD^:6xMyc/UAS-cherry-RNAi* |
|  | *UAS-bsk^DN^/+; sal>UAS-GFP/+; p53-B^ΔDBD^:6xMyc:/+* |
| Figure S6 | Genotype |
| S6A | *+/*(*+*)*; nub>UAS-GFP/+; +/+* |
|  | *+/*(*+*)*; nub>UAS-GFP/+; UAS-6xMyc:p53-E/cherry-RNAi* |
|  | *+/*(*+*)*; nub>UAS-GFP/UAS-miRHG; +/UAS-6xMyc:p53-E* |
|  | *UAS-bsk^DN^/+; nub>UAS-GFP/UAS-miRHG; +/UAS-6xMyc:p53-E* |
| Figure S7 | Genotype |
| S7A | *+/*(*+*)*; sal>, rpr-p53^RE^-GFP/+; UAS-hp53:6xMyc/+* |
|  | *+/*(*+*)*; sal>, hid-p53^RE^-GFP/+; UAS-hp53:6xMyc/+* |
|  | *+/*(*+*)*; sal>GFP, TRE-RFP/+; UAS-hp53:6xMyc/+* |
|  | *+/*(*+*)*; sal>GFP, TRE-RFP/+; UAS-hp53^ΔDBD^:6xMyc/+* |
| S7B | *+/*(*+*)*; sal>UAS-GFP/+; UAS-hp53:6xMyc/UAS-cherry-RNAi* |
|  | *UAS-bsk^DN^/+; sal>UAS-GFP/+; UAS-hp53:6xMyc/+* |
|  | *+/*(*+*)*; sal>UAS-GFP/+; UAS-hp53^ΔDBD^:6xMyc/UAS-cherry-RNAi* |
|  | *UAS-bsk^DN^/+; sal>UAS-GFP/+; UAS-hp53^ΔDBD^:6xMyc/+* |
